# Inhibition of Aminoglycoside 6′-*N*-acetyltransferase Type Ib (AAC(6′)-Ib): Structure–Activity Relationship of Substituted Pyrrolidine Pentamine Derivatives as Inhibitors

**DOI:** 10.3390/biomedicines9091218

**Published:** 2021-09-14

**Authors:** Kenneth Rocha, Jesus Magallon, Craig Reeves, Kimberly Phan, Peter Vu, Crista L. Oakley-Havens, Stella Kwan, Maria Soledad Ramirez, Travis LaVoi, Haley Donow, Prem Chapagain, Radleigh Santos, Clemencia Pinilla, Marc A. Giulianotti, Marcelo E. Tolmasky

**Affiliations:** 1 Center for Applied Biotechnology Studies, Department of Biological Science, College of Natural Sciences and Mathematics, California State University Fullerton, Fullerton, CA 92831, USA; kenneth.rocha@csu.fullerton.edu (K.R.); jesusmagallon@fullerton.edu (J.M.); wizard5424@csu.fullerton.edu (C.R.); kkphan@csu.fullerton.edu (K.P.); vupeter8@csu.fullerton.edu (P.V.); cristalee0810@csu.fullerton.edu (C.L.O.-H.); shkwan@csu.fullerton.edu (S.K.); msramirez@fullerton.edu (M.S.R.); 2 Center for Translational Science, Florida International University, Port St. Lucie, FL 34987, USA; tlavoi@fiu.edu (T.L.); hdonow@fiu.edu (H.D.); cpinilla@fiu.edu (C.P.); mgiulian@fiu.edu (M.A.G.); 3 Department of Physics, Florida International University, Miami, FL 33199, USA; chapagap@fiu.edu; 4 Biomolecular Sciences Institute, Florida International University, Miami, FL 33199, USA; 5 Department of Mathematics, Nova Southeastern University, Fort Lauderdale, FL 33314, USA; radleigh@nova.edu

**Keywords:** aminoglycoside resistance, structure–activity relationship, aminoglycoside-modifying enzymes, acetyltransferase, *Acinetobacter*

## Abstract

The aminoglycoside 6′-*N*-acetyltransferase type Ib (AAC(6′)-Ib) is a common cause of resistance to amikacin and other aminoglycosides in Gram-negatives. Utilization of mixture-based combinatorial libraries and application of the positional scanning strategy identified an inhibitor of AAC(6′)-Ib. This inhibitor’s chemical structure consists of a pyrrolidine pentamine scaffold substituted at four locations (R1, R3, R4, and R5). The substituents are two *S*-phenyl groups (R1 and R4), an *S*-hydroxymethyl group (R3), and a 3-phenylbutyl group (R5). Another location, R2, does not have a substitution, but it is named because its stereochemistry was modified in some compounds utilized in this study. Structure–activity relationship (SAR) analysis using derivatives with different functionalities, modified stereochemistry, and truncations was carried out by assessing the effect of the addition of each compound at 8 µM to 16 µg/mL amikacin-containing media and performing checkerboard assays varying the concentrations of the inhibitor analogs and the antibiotic. The results show that: (1) the aromatic functionalities at R1 and R4 are essential, but the stereochemistry is essential only at R4; (2) the stereochemical conformation at R2 is critical; (3) the hydroxyl moiety at R3 as well as stereoconformation are required for full inhibitory activity; (4) the phenyl functionality at R5 is not essential and can be replaced by aliphatic groups; (5) the location of the phenyl group on the butyl carbon chain at R5 is not essential; (6) the length of the aliphatic chain at R5 is not critical; and (7) all truncations of the scaffold resulted in inactive compounds. Molecular docking revealed that all compounds preferentially bind to the kanamycin C binding cavity, and binding affinity correlates with the experimental data for most of the compounds evaluated. The SAR results in this study will serve as the basis for the design of new analogs in an effort to improve their ability to induce phenotypic conversion to susceptibility in amikacin-resistant pathogens.

## 1. Introduction

A growing number of Gram-negative pathogens are rapidly acquiring resistance to most, and in some cases all, antibiotics in use [1]. As a consequence, treatment of severe infections caused by multidrug-resistant (MDR) bacteria is becoming more complicated and prohibitively expensive [2]. The magnitude of the problem is illustrated by the inclusion of MDR *Acinetobacter baumannii* and other Gram negatives such as *Klebsiella pneumoniae* and *Pseudomonas aeruginosa* as “Priority 1:Critical” in the World Health Organization Priority Pathogens list for Research and Development of new antibiotics [3]. The urgency to develop new treatments against these pathogens requires not only the design of novel antibiotics but also the finding of adjuvants that, in combination with existing drugs, circumvent the resistance [4]. This latter strategy extends the useful life of antibiotics already in use, but that are becoming ineffective due to the dissemination of resistance traits. This strategy has been successful for β-lactams, in which case several β-lactam/β-lactamase inhibitor formulations are currently in use [5,6]. On the other hand, the identification or design of inhibitors of resistance to other classes of antibiotics has not progressed beyond the research laboratory.

Aminoglycosides are bactericidal antibiotics that interfere with translational fidelity, producing proteins with incorrect primary sequences that lead to multiple toxic physiological effects and, ultimately, cell death [7,8,9]. These antibiotics have been instrumental in treating life-threatening infections caused by Gram-negative and, in combination with other antimicrobials, Gram-positive bacteria [7,10]. Although bacteria have developed various mechanisms to resist aminoglycosides, enzymatic inactivation is the most prevalent in the clinical setting [8,11,12]. There are numerous reports of compounds that interfere with the inactivation of the antibiotic molecule by different molecular mechanisms or enhance the cellular uptake [8,13,14,15,16,17,18,19,20,21,22,23,24,25]. However, despite their demonstrated activity, none of them could be turned into formulations for clinical use.

The aminoglycoside 6′-*N*-acetyltransferase type Ib (AAC(6′)-Ib) causes resistance to amikacin and other aminoglycosides in Gram-negative bacteria [12]. Since this is the most common enzyme among AAC(6′)-I-producing Gram-negative pathogens [8,9,12,26,27], it was selected as the target in the quest for inhibitors that, in combination with amikacin, could be used to treat resistant infections. In particular, recovering susceptibility to amikacin could help control those caused by strains resistant to carbapenems, which are antimicrobials of last resort for treatment of several MDR infections [6]. We have recently identified an inhibitor of AAC(6′)-Ib using mixture-based combinatorial libraries and the positional scanning strategy [24,28]. The compound consists of a pyrrolidine pentamine scaffold with two S-phenyl groups, an S-hydroxymethyl group, and a 3-phenylbutyl group at the positions shown in Table 1. The structure–activity relationship (SAR) study described in this article was carried out to better understand this compound’s properties as an inhibitor of AAC(6′)-Ib and design related compounds with more robust activity.

## 2. Materials and Methods

### 2.1. Bacterial Strain and Cultures

*A. baumannii* A155 was originally isolated from a urinary sample at a hospital in Buenos Aires, Argentina [29]. It belongs to the clonal complex 109, it is multiple drug resistant, and it naturally carries *aac(6’)-Ib* [30,31]. This strain was utilized in this work because inhibition of expression of *aac(6’)-Ib* by an antisense oligonucleotide analog results in complete obliteration of the resistant phenotype [30]. Routine cultures were carried out in Lennox L broth (1% tryptone, 0.5% yeast extract, 0.5% NaCl), and 2% agar was added in the case of solid medium. For determination of levels of resistance to amikacin, the culture medium used was Mueller-Hinton broth. 

### 2.2. Synthesis of Small Molecule Compounds

All molecules screened were synthesized at the Torrey Pines Institute for Molecular Studies (now the Center for Translational Science at Florida International University) as previously described [24]. Briefly, a polyamide scaffold was synthesized on a solid support using standard Boc chemistry, the amide residues were reduced with borane, and the compounds were removed from the solid support (Figure S1).

### 2.3. High-Performance Liquid Chromatography (HPLC) Purification 

All purifications were performed on a Shimadzu Prominence preparative HPLC system consisting of LC-8A binary solvent pumps, an SCL-10A system controller, a SIL-10AP auto sampler, and an FRC-10A fraction collector. A Shimadzu SPD-20A UV detector set to 254 nm was used for detection. Chromatographic separations were obtained using a Phenomenex Gemini C18 preparative column (5 μm, 150 mm × 21.5 mm i.d.) with a Phenomenex C18 column guard (5 μm, 15 mm × 21.2 mm i.d.). Prominence prep software was used to set all detection and collection parameters. The mobile phases for HPLC purification were HPLC grade obtained from Sigma-Aldrich and Fisher Scientific. The mobile phase consisted of a mixture of acetonitrile/water (both with 50 mM acetic acid). The initial setting for separation was 2% acetonitrile, which was held for 2 min, then the gradient was linearly increased to 6% acetonitrile over 4 min. The gradient was then linearly increased to 35% acetonitrile over 29 min. The HPLC system was set to automatically flush and re-equilibrate the column after each run for a total of four column volumes. The total flow rate was set to 15 mL/min, and the total injection volume was set to 2 mL. The fractions corresponding to the desired product were then combined and lyophilized.

### 2.4. Liquid Chromatography–Mass Spectrometry (LCMS) Analysis of Purified Material

The purity and identity of compounds were verified using a Shimadzu 2010 LCMS system consisting of an LC-20AD binary solvent pump, a DGU-20A degasser unit, a CTO-20A column oven, and a SIL-20A HT auto sampler. A Shimadzu SPD–M20A diode array detector scanned the spectrum range of 190–400 nm during the analysis. Chromatographic separations were obtained using a Phenomenex Luna C18 analytical column (5 μm, 150 mm × 4.6 mm i.d.) with a Phenomenex C18 column guard (5 μm, 4 × 3.0 mm i.d.). All equipment was controlled and integrated by Shimadzu LCMS solutions software version 3. Mobile phase A for LCMS analysis was LCMS-grade water, and mobile phase B was LCMS-grade acetonitrile obtained from Sigma-Aldrich and Fisher Scientific (both with 0.1% formic acid for a pH of 2.7). The initial setting for analysis was 5% acetonitrile (*v*/*v*), and then linearly increased to 95% acetonitrile over 14 min. The gradient was then held at 95% acetonitrile for 2 min before being linearly decreased to 5% over 2 min and held until stop for an additional 2 min. The total run time was 20 min, and the total flow rate was 0.5 mL/min. The column oven and flow cell temperature for the diode array detector was 40 °C. The auto sampler was at room temperature, and a 5 μL aliquot was injected for analysis. Pertinent information on characterization and degree of purification of the compounds can be found in Figures S2 and S3.

### 2.5. Initial Growth Inhibition Assays

Growth inhibition of the compounds was initially determined measuring OD_600_ after 20 h of growth. The data are expressed in percent inhibition based on the OD_600_ measurements (Table 1). Amikacin and potential inhibitor concentrations were selected based on checkerboards analyses done on the original **2637.001** compound [24]. Each compound was tested in five separate experiments by duplicate. The average and standard error of the mean for n = 10 percentage growth inhibition values of each compound were calculated. The *p*-value for testing the difference in growth inhibitory activity between a given compound and **2637.001** was calculated using a two-sample t-test with Bonferroni–Holm correction. A *p*-value of less than 0.05 was considered significant.

### 2.6. Modeling 

The structures of the compounds were converted to 3D structures with added polar hydrogen bonds using Open Babel [32]. The structure of AAC(6’)-Ib complexed with kanamycin C and AcetylCoA [27] was obtained from the protein data bank (PDB 1V0C). The AAC(6′)-Ib protein with kanamycin C removed and the compounds were prepared in the pdqt format using AutoDockTools 4.2 [33]. A cavity in the kanamycin C binding region of the protein was selected as the target site for virtual screening. Vina from AutoDockTools 4.2 [33] was used to perform docking and screening. The docking scores were sorted and ranked based on their predicted binding energies. LigPlot+ [34] was used to generate a 2D ligand–protein interaction map. PyMol 2.3 (Schrodinger) was used for visualization and rendering.

### 2.7. Checkerboard Assays 

Checkerboard assays were performed in Mueller-Hinton broth with variable concentrations of the compound to be tested (0, 4, 8, 16, and 24 μM) and amikacin (0, 8, 16, 32, and 64 μg/mL) in microtiter plates using the BioTek Synergy 5 microplate reader (BioTek Synergy 5) as described before [24]. All compounds that did not show a significant reduction (*p* < 0.01, two-sample t-test versus compound **2637.001**) in the initial screening were chosen for checkerboard assay. Since there is a chance that the testing compounds have some residual antimicrobial activity, data were analyzed using an approach that quantifies exact levels of synergy [24,35]. The model considers that amikacin and the compounds to be tested have independent antimicrobial mechanisms of action. The percent activity of the mixture of the two chemicals was modeled as:

%_*amikacin & compound*_(x_1_,x_2_) = %_*amikacin*_(x_1_) + % _*compound*_(x_2_) − %_*amikacin*_(x_1_).% _*compound*_(x_2_)

In this equation, x_1_ and x_2_ are the concentrations of amikacin and tested compound, respectively. To calculate the effective percent activity of the antibiotic alone at a given concentration, after accounting for compound activity the previous equation can be rearranged as follows:

Eff%*_amikacin_*(x_1_) = (%*_amikacin & compound_*(x_1_,x_2_) − %_*compound*_(x_2_))/(1 − %_*compound*_(x_2_))

This methodology informs the actual change in amikacin resistance levels. Four checkerboard assays were performed for each compound, and the above methodology was applied to the median of the four values at each dose combination.

Once applied to the checkerboard data, a 95% confidence interval for the mean effective concentration of amikacin to achieve 50% inhibition (IC_50_) at each dose of potentiating compound was determined using standard curve fitting of Hill’s equation.

## 3. Results

### 3.1. Synthesis and Preliminary Analysis of Analogs to Compound ***2637.001***

The recent identification of an inhibitor of the AAC(6′)-Ib opened new possibilities to formulate combinations with aminoglycosides to treat resistant infections. This compound’s chemical structure consists of a pyrrolidine pentamine scaffold substituted with two *S*-phenyl groups, an *S*-hydroxymethyl group, and a 3-phenylbutyl group at the positions R1, R3, R4, and R5, respectively (compound **2637.001**, Table 1). (Note that **2637.001** was referred to as compound **2155-206** in the previous publication [24]). A SAR set of experiments with a series of compound **2637.001** analogs was initiated to gain insights into the different chemical groups’ contribution to the AAC(6′)-Ib inhibitory effect. The primary goal of this preliminary SAR study was to assess the relative importance of each specific functionality and stereochemistry as well as determine the minimal pharmacophore needed. Therefore, compounds were designed with a single substitution at each of the R positions or truncation of a specific scaffold fragment (Table 1). The effect of the addition of each compound at 8 µM concentration to 16 µg/mL amikacin-containing medium on growth of the *aac(6′)-Ib*-harboring *A. baumannii* A155 strain was tested. The concentration of amikacin was chosen based on previous studies showing that this strain grows in the breakpoint concentration 16 µg/mL amikacin [36]. Bacterial growth was assessed measuring OD_600_ after 20 h of incubation, which is a time when the cultures were already in stationary phase, and the values were used to calculate the percentage of inhibition of resistance (Table 1). The growth curve of *A. baumannii* A155 cultured in Mueller-Hinton broth with no additions, addition of amikacin, or addition of amikacin plus compound 2637.001 is shown in Figure S4. The different degrees of growth inhibition observed in these assays indicate that the structural changes in the analogs with respect to the compound **2637.001** must affect the AAC(6′)-lb inhibitory efficacy.

The importance of the *S*-phenyl at the R1 position was assessed by modifying the chemical group or the stereochemistry (Table 1). In compound **2637.002**, the aromatic phenyl group was removed, leaving an *S*-methyl functionality, and in compound **2637.003**, the phenyl moiety was separated from the backbone by the addition of a methylene group (Table 1). In both cases, the *S* conformation was maintained. In compound **2637.020**, the *S*-phenyl was replaced by an *R*-phenyl functionality changing only the stereochemistry. Table 1 shows that replacing the aromatic functionality with a methyl group significantly reduced the percentage of inhibition (hereafter referred to as inhibitory activity) with respect to that observed when compound **2637.001** is tested (62% vs. 18%). Interestingly, a methylene group placed between the scaffold and the phenyl functionality (benzyl) (**2637.003**) also affected the inhibitory activity, reducing it to 20%. This reduction in inhibitory activity could be due to the loss of the aromatic group’s ability to interact or stabilize the interaction with the appropriate region of AAC(6′)-Ib. Conversely, the absolute stereochemistry at this position does not appear to be critical as the *S* and *R* conformations produced similar inhibitory activities (**2637.001**, 62% vs. **2637.020**, 73%).

Position R2 was not originally considered a location for addition of functionalities. However, in this study the relative importance of the stereochemistry at this position was assessed (Table 1). An analog, **2637.021**, was synthesized, where the R2 stereocenter was modified from *S* to *R*. This change resulted in a compound with a significantly reduced capability to inhibit resistance to amikacin (**2637.001**, 62% vs. **2637.021**, 28%). This result demonstrated that absolute stereochemistry plays a crucial role at this position.

At the R3 position, which has an *S*-hydroxymethyl in **2637.001**, analogs that modify the functionality or the stereochemistry were assessed (Table 1). Compound **2637.005** differs from **2637.001** in the stereoconfiguration, which was altered from *S* to *R*. This compound was used to determine the relative importance of the absolute conformation at this position. Table 1 shows that the modification significantly impacted the inhibitory activity (**2637.001**, 62% vs. **2637.005**, 24%), probably by impeding the appropriate interaction between the hydroxy moiety and the target.

The two other analogs with modifications at the R3 positions were compounds **2637.004** and **2637.019**, in which the hydroxy group was eliminated (Table 1). The conformation was maintained in the former and changed to *R* in the latter. It was interesting that when the stereochemistry of the parent compound was preserved, the inhibitory activity was slightly reduced (**2637.001**, 62% vs. **2637.004**, 39%), suggesting that the hydroxyl group is needed and likely is involved in hydrogen bonding between the parent compound **2637.001** and AAC(6′)-Ib. However, when the stereochemistry was changed, the compound showed a comparable capacity for inhibiting growth to the parent compound (**2637.019**, 74% vs. **2637.001**, 62%). This result suggests a different binding motif for this analog that does not require the hydroxyl group present in the parent compound.

The *S*-phenyl group at the R4 position in the compound **2637.001** was replaced by *S*-methyl (**2637.006**) or *R*-phenyl (**2637.022**) groups (Table 1). The results obtained when adding compound **2367.006** or **2637.022** to the culture medium show the importance of the aromatic functionality and the S conformation at the R4 position, respectively. The reduction in levels of inhibition of resistance indicates that both modifications had significant effects suggesting an important role of the phenyl moiety and its steric configuration. 

Modifications at the R5 position resulted in three groups of analogs (Table 1). The first set was designed to examine the effect of removing the aromatic phenyl group and replacing it with aliphatic groups with various carbon chain lengths. In compound **2637.008**, the phenyl group was removed from the 3-position of the butyl group. The phenyl group was removed in compounds **2637.007** and **2637.010**, and the aliphatic chains were modified to contain either two or five carbons, respectively. The compounds **2637.007** and **2637.010**, in which the phenyl group was removed, and the carbon chain length was reduced or lengthened with respect to compound **2637.001**, exhibited similar levels of inhibition of resistance to the parent compound (**2637.001** 62%, **2637.007** 60%, and **2637.010** 71%). These results suggest that the phenyl functionality is not essential. In the case that an aliphatic functionality is used at this position, there are potential options regarding sizing, branching, and additional substituents, thus allowing for lipophilic optimization as needed.

The second set of analogs is characterized by modifications in the location of the phenyl group on the butyl carbon chain (Table 1). In compounds **2637.011** and **2637.012**, the phenyl group is bound to the second or the fourth carbon, respectively. Analog **2637.011** produced a similar inhibitory effect compared to the parent compound (**2637.001**, 62% vs. **2637.011**, 66%). Having comparable activities, analog **2637.011** presents the added benefit that by placing the substituent at position 4, the undefined stereocenter on the parent compound is eliminated. The result obtained with analog **2637.012** had lower but still evident activity (40%), suggesting that the phenyl group located at the R5 position can be moved without a drastic loss of activity.

The last set includes compounds **2637.013** and **2637.014** (Table 1). Compound **2637.013** maintains the phenyl group on the terminal carbon, but it is bound to a shorter aliphatic carbon chain (propyl). This conformation examines the effects of eliminating the last carbon moiety at the R5 position of the parent compound and provides an analog without a stereocenter in this position. It was encouraging to note that this analog also maintained activity (62%) as it allows for another compound where the undefined stereochemistry is eliminated at the R5 position. Compound **2637.014** builds on **2637.013** by examining the effect of introducing an aromatic heterocyclic moiety at the R5 position. While this compound had lower activity (46%), it could suggest that a heterocyclic moiety can be introduced at this position.

The last set of compounds is composed of truncated analogs (Table 1). This set was utilized to assess the minimal pharmacophore needed to preserve inhibitory activity when scanning from the R1 to the R5 direction. In compounds **2637.015** and **2637.016**, the R5 functionality was eliminated, and compound **2637.016** was reduced further by removing the phenyl group of the R4 functionality. Compound **2637.017** was further reduced, eliminating the primary amine and *S*-methyl groups. Finally, compound **2637.018** was designed to lack both the R4 and R5 groups from the parent compound. None of the analogs from this set produced significant inhibitory activity in the primary assay, suggesting that the entire scaffold is essential. However, future studies will be necessary to confirm the essentiality of other scaffold regions.

### 3.2. Molecular Docking

**Figure 1:**
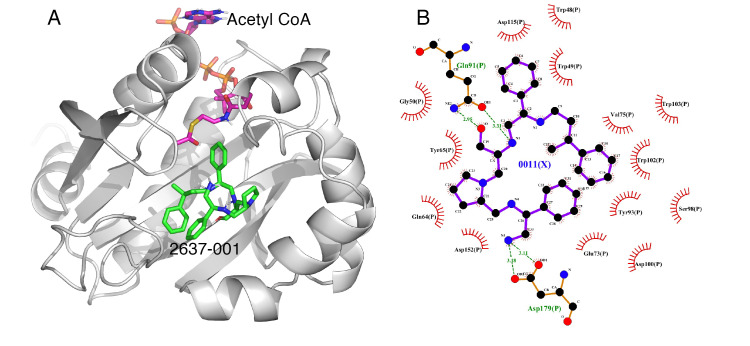
(**A**) The complex of **2637.001** and AAC(6′)-Ib obtained from molecular docking. The bound acetyl CoA is also shown. (**B**) Interaction map of the ligand in its binding site of the AAC(6′)-Ib receptor. The map shows the hydroxyl functionality interaction with Gln91 and the primary amine of **2637.001** hydrogen bonding with Asp179. The pictures show the top-ranked, lowest-energy conformation.

The data from the SAR study suggest that specific changes in absolute stereochemistry or elimination of key functional groups can affect the compound’s ability to enhance the amikacin antibacterial response in the primary screening assay (Table 1). The binding poses of the twenty compounds in Table 1 as well as amikacin against AAC(6′)-Ib were investigated to explore potential critical interactions responsible for these changes in inhibitory efficacy. A blind docking revealed that the compounds all preferentially bind to the kanamycin C binding cavity and, therefore, this site is considered as the target site for docking. To incorporate the flexibility of the sidechains around the target site, flexible docking was performed with W49, Y65, E73, V75, Q91, Y93, S98, D100, W103, D115, D152, and D179 as the flexible residues. The screening revealed that **2637.001** is one of the top compounds to bind AAC(6′)-Ib effectively and, based on the Delta G value obtained from docking, it is predicted to bind more effectively than amikacin (Table 1). Figure 1A shows the AAC(6′)-Ib–compound complex, showing the binding pose of **2637.001** in the kanamycin C binding site. This is the same binding site predicted for amikacin (Figure S5). A 2D map of the ligand in the binding site shows that Q91 and D179 make hydrogen-bond interactions, in addition to other residues involved in hydrophobic interactions (Figure 1B).

**Figure 2:**
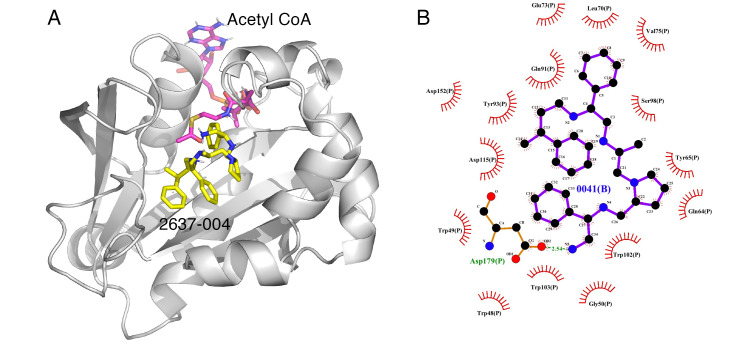
(**A**) The complex of **2637.004** and AAC(6′)-Ib obtained from molecular docking. The bound acetyl CoA is also shown. (**B**) Interaction map of the ligand in its binding site of the AAC(6′)-Ib receptor. The primary amine of **2637.004** maintains a hydrogen bond interaction with Asp179. The pictures show the top-ranked, lowest-energy conformation.

**Figure 3:**
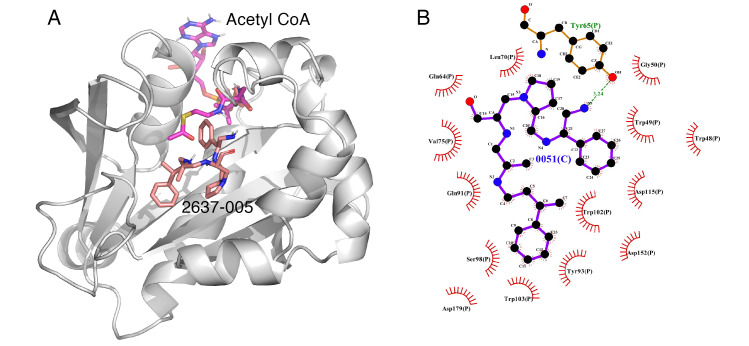
(**A**) The complex of **2637.005** and AAC(6′)-Ib obtained from molecular docking. The bound acetyl CoA is also shown. (**B**) Interaction map of the ligand in its binding site of the AAC(6′)-Ib receptor. Compound **2637.005** adopts a different orientation in the pocket and now the primary amine interacts with the phenol group of Tyr65 and the hydroxyl group is no longer close enough to hydrogen bond with Gln64. The pictures show the top-ranked, lowest-energy conformation.

**Figure 4:**
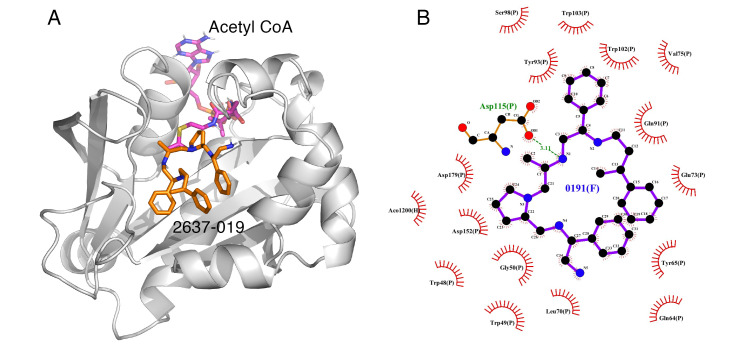
(**A**) The complex of **2637.019** and AAC(6′)-Ib obtained from molecular docking. The bound actyl CoA is also shown. The figure shows a potential for intramolecular pi stacking between the R1 and R5 phenyl groups. (**B**) Interaction map of the ligand in its binding site of the AAC(6′)-Ib receptor. Compound **2637.019** still maintains a hydrogen bond interaction with Asp115. The pictures show the top-ranked, lowest-energy conformation.

Some correlations were found when comparing the predicted binding efficacies and poses to the screening data and SAR observations (Table 1, Delta G, and Figure 1, Figure 2, Figure 3 and Figure 4). For example, looking in detail at the R3 position analogs (**2637.001**, **2637.004**, **2637.005**, **2637.019**), some trends from the docking study support the SAR observations noted previously. Compound **2637.004** eliminates the hydroxyl group from the parent compound, which is shown to hydrogen bond with the target protein (Figure 1). Compound **2637.004** is predicted to have a slightly lower binding efficiency than **2637.001** (Table 1), and the binding pose (Figure 2A,B) shows that it now only interacts with the Asp179 residue, thus providing a rationale for the reduction in inhibitory activity noted for **2637.004** (Table 1). In compound **2637.005**, the absolute stereochemistry of the hydroxy group is changed. This modification significantly affected the inhibitory activity and predicted binding efficacy (Table 1). The 2D map (Figure 3A,B) shows that the compound has a preferential reorientation in the binding pocket so that the hydroxy group no longer interacts with the target protein. Additionally, the R1 and R5 phenyl rings on **2637.005** no longer interact through pi stacking, which also may contribute to the loss of inhibitory activity and predicted binding efficacy. In compound **2637.019**, the hydroxy group at the R3 position was eliminated and the absolute stereochemistry at this position was modified. This compound maintained inhibitory activity (Table 1, % inhibition) and was predicted to have slightly less binding efficiency than the parent compound (Table 1, Delta G). Looking at the 2D map (Figure 4A,B), it appears that **2637.019** potentially compensates for the loss of the hydroxyl group by maintaining a hydrogen bond interaction with Asp115 as well as increasing intramolecular pi stacking between the R1 and R5 benzyl groups.

### 3.3. Potentiation

A more precise analysis of the ability of the analogs listed in Table 1 that did not show a significant deviation (*p* < 0.01) in inhibitory activity from **2637.001** was carried out using checkerboard assays. The percent growth inhibition results at all doses are shown in Figure S6 and the analyzed results in Table 2. The checkerboard assays confirmed that compounds **2637.020**, **2637.007**, **2637.010**, **2637.011**, and **2637.013** do not significantly differ in potentiation behavior from **2637.001**. For example, amikacin has a potentiated IC_50_ of 9.5 μM (95% C.I. (7.5, 11.4)) in the presence of 8 μM of **2637.020**, versus equivalent values of 8.5 μM (95% C.I. (5.5, 11.5)) for **2637.001**. Similar overlaps in confidence intervals for the other compounds are shown in Table 2.

Three compounds, **2637.004**, **2637.012**, and **2637.014**, showed a slight reduction in the initial screening, and were confirmed to have significantly lower potentiating ability in the checkerboard assay. For example, amikacin has a potentiated IC_50_ of 20.6 μM (95% C.I. (14.9, 26.2)) in the presence of 8 μM of **2637.004**, versus equivalent values of 8.5 μM (95% C.I. (5.5, 11.5)) for **2637.001**. Similar non-overlaps in confidence intervals for the other compounds are shown in Table 2.

Compound **2637.019** showed antimicrobial activity on its own when tested in the checkerboard assay, with a median percent inhibition of 19.1% at 16 μM with no amikacin. No other compound tested in the checkerboard assay exceeded 6.3% inhibition at this dose (see Figure S6). The adjusted values after discounting the antimicrobial activity showed that the compound **2637.019** did not show any consistent difference in potentiation ability versus **2637.001** (as seen in the overlap of confidence intervals in Table 2).

## 4. Discussion

The quest to confront the antibiotic resistance crisis, one of the top threats to human health, requires multifactorial approaches to stop the selection and dissemination of resistant pathogens, design or discover new antimicrobials, and devise strategies to prolong drugs’ useful life [2,4,37]. A very successful approach to achieve this latter objective in the case of β-lactams was developing inhibitors of β-lactamases that are administered in combination with the antibiotic to eliminate the pathogen’s ability to hydrolyze the antibiotic [5,6]. Unfortunately, such a successful alternative has not yet been fully developed for aminoglycosides. Despite significant efforts, no formulations that combine an aminoglycoside and an inhibitor of the resistance have been approved for human use [4,38]. Although there are many mechanisms and variations by which bacteria resist aminoglycosides, the presence of AAC(6′)-Ib in the majority of Gram-negative amikacin-resistant clinical strains implies that the search to find inhibitors that permit their use in a significant number of infections may not be as insurmountable as it seems [4,8,39]. In particular, effective inhibition of AAC(6′)-Ib-mediated resistance would restore the efficiency of amikacin as a treatment of the currently most dangerous MDR carbapenem-resistant infections [40,41].

Using mixture-based combinatorial libraries and the positional scanning strategy led to identification of an inhibitor of AAC(6′)-Ib that, when supplied in combination with amikacin, overcame resistance in several bacteria [24]. The chemical structure of this compound consists of a pyrrolidine pentamine scaffold with two S-phenyl groups, an S-hydroxymethyl group, and a 3-phenylbutyl group at the positions shown in Table 1. In this study, a series of analogs were analyzed to gain insights into the role that parts of the scaffold, stereochemistry, and functional groups decorating the scaffold play in driving inhibitory activity and ultimately potentiation. For most of the positions (R2, R3, and R4), the absolute stereochemistry of the parent compound was critical for maintaining the inhibitory activity of the compound. The truncation studies showed that the complete pyrrolidine pentamine scaffold is necessary for maintaining inhibitory activity (though further studies with truncations in the opposite direction remain to conclusively determine if the entire scaffold is necessary). At most of the positions (R1, R3, and R5), there was at least a single point substitution analog that maintained the level of inhibitory activity and ultimately the ability to potentiate amikacin at levels comparable to the parent **2637.001** compound.

A molecular docking approach showed that the compounds compete for the same binding site as amikacin against AAC(6′)-Ib, further validating the potential mechanism by which the compounds potentiate amikacin. Through this same molecular docking approach, it was evident that some of the compounds with better inhibitory activity have more binding interactions with AAC(6′)-Ib than those with weaker inhibitory activity.

Taken together, the results shown in this study validate the concept that inhibiting AAC(6′)-Ib is a potential avenue to preserve the antimicrobial efficacy of amikacin against Gram-negative amikacin-resistant clinical strains. A medicinal chemistry approach that incorportates molecular modeling to explore additional analogs based on the pyrrolidine pentamine scaffold holds promise to identify clinical candidates.

## Figures and Tables

**Table 1:** Properties of **2637.001** analogs.

Compound Name	Chemical Structure	Functionalities	%Inhibition (Average, n = 10)	Standard Error	Delta G Kcal/mL (Average, n = 3)
**2637.001**	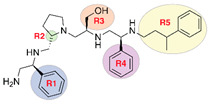	R1: *S*-phenyl R2: *S*-pyrrolidine R3: *S*-hydroxymethyl R4: *S*-phenyl R5: 3-phenylbutyl	62	4	−9.5 ± 0.1
		**R1 analogs**			
**2637.002**	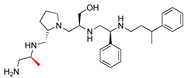	R1: *S*-methyl R2: *S*-pyrrolidine R3: *S*-hydroxymethyl R4: *S*-phenyl R5: 3-phenylbutyl	18 *	2	−8.7 ± 0.1
**2637.003**	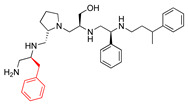	R1: *S*-benzyl R2: *S*-pyrrolidine R3: *S*-hydroxymethyl R4: *S*-phenyl R5: 3-phenylbutyl	20 *	3	−8.0 ± 0.1
**2637.020**	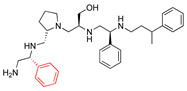	R1: *R*-phenyl R2: *S*-pyrrolidine R3: *S*-hydroxymethyl R4: *S*-phenyl R5: 3-phenylbutyl	73	4	−8.5 ± 0.3
		**R2 analogs**			
**2637.021**	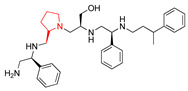	R1: *S*-phenyl R2: *R*-pyrrolidine R3: *S*-hydroxymethyl R4: *S*-phenyl R5: 3-phenylbutyl	28 *	4	−9.5 ± 0.3
		**R3 analogs**			
**2637.004**	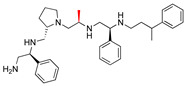	R1: *S*-phenyl R2: *S*-pyrrolidine R3: *S*-methyl R4: *S*-phenyl R5: 3-phenylbutyl	39	6	−9.2 ± 0.1
**2637.005**	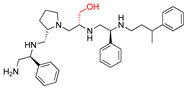	R1: *S*-phenyl R2: *S*-pyrrolidine R3: *R*-hydroxymethyl R4: *S*-phenyl R5: 3-phenylbutyl	24 *	2	−8.2 ± 0.1
**2637.019**	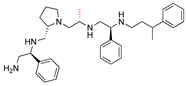	R1: *S*-phenyl R2: *S*-pyrrolidine R3: *R*-methyl R4: *S*-phenyl R5: 3-phenylbutyl	74	9	−9.2 ± 0.1
		**R4 analogs**			
**2637.006**	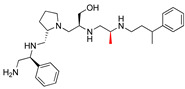	R1: *S*-phenyl R2: *S*-pyrrolidine R3: *S*-hydroxymethyl R4: *S*-methyl R5: 3-phenylbutyl	23 *	2	−7.9 ± 0.2
**2637.022**	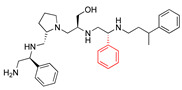	R1: *S*-phenyl R2: *S*-pyrrolidine R3: *S*-hydroxymethyl R4: *R*-phenyl R5: 3-phenylbutyl	28 *	4	−9.6 ± 0.1
		**R5 analogs**			
**2637.007**	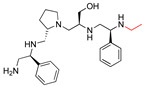	R1: *S*-phenyl R2: *S*-pyrrolidine R3: *S*-hydroxymethyl R4: *S*-phenyl R5: ethyl	60	1	−9.5 ± 0.1
**2637.008**	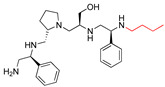	R1: *S*-phenyl R2: *S*-pyrrolidine R3: *S*-hydroxymethyl R4: *S*-phenyl R5: butyl	17 *	2	−9.4 ± 0.1
**2637.010**	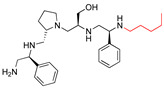	R1: *S*-phenyl R2: *S*-pyrrolidine R3: *S*-hydroxymethyl R4: *S*-phenyl R5: pentyl	71	2	−9.1 ± 0.2
**2637.012**	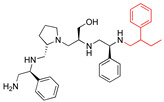	R1: *S*-phenyl R2: *S*-pyrrolidine R3: *S*-hydroxymethyl R4: *S*-phenyl R5: 2-phenylbutyl	40	6	−8.5 ± 0.2
**2637.011**	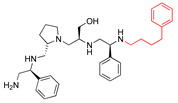	R1: *S*-phenyl R2: *S*-pyrrolidine R3: *S*-hydroxymethyl R4: *S*-phenyl R5: phenylbutyl	66	6	−8.1 ± 0.2
**2637.013**	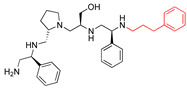	R1: *S*-phenyl R2: *S*-pyrrolidine R3: *S*-hydroxymethyl R4: *S*-phenyl R5: phenylpropyl	62	3	−9.1 ± 0.1
**2637.014**	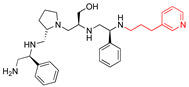	R1: *S*-phenyl R2: *S*-pyrrolidine R3: *S*-hydroxymethyl R4: *S*-phenyl R5: (pyridiin−3-yl)propyl	46	3	−9.1 ± 0.2
		**Truncation Analogs**			
**2637.015**	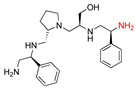	R1: *S*-phenyl R2: *S*-pyrrolidine R3: *S*-hydroxymethyl R4: *S*-phenyl R5: hydrogen	26 *	3	−8.7 ± 0.1
**2637.016**	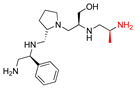	R1: *S*-phenyl R2: *S*-pyrrolidine R3: *S*-hydroxymethyl R4: *S*-methyl R5: hydrogen	20 *	3	−8.2 ± 0.1
**2637.017**	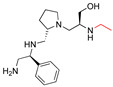	R1: *S*-phenyl R2: *S*-pyrrolidine R3: *S*-hydroxymethyl R4: modified to ethyl R5: nothing	21 *	3	−8.4 ± 0.1
**2637.018**	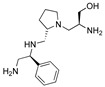	R1: *S*-phenyl R2: *S*-pyrrolidine R3: *S*-hydroxymethyl R4: nothing R5: nothing	17 *	2	−8.2 ± 0.1

* *p*-Value < 0.01, Two-Sample T-Test with Bonferroni–Holm Correction when compared to **2637.001**. The percent inhibition of 16 μg/mL amikacin with no additions is 22.7 ± 7.8. Delta G Kcal/mL values are the binding scores obtained from molecular docking and reported as averages of the top three docking scores. For comparison, amikacin docking gave a Delta G of −8.2 ± 0.2.

**Table 2:** Summary of checkerboard assays.

		Compound Dose (μM)														
		0			4			8			16			24		
Compound Name	R Group Modified	IC_50_ μM	95% CI		IC_50_ μM	95% CI		IC_50_ μM	95% CI		IC_50_ μM	95% CI		IC_50_ μM	95% CI	
**2637.001**	NA	**24.9**	18.2	31.5	**12.9**	11.1	14.7	**8.5**	5.5	11.5	**0.8**	0.2	1.3	**0.2**	0.2	1.3
**2637.020**	R1	**23.6**	21.2	26.0	**12.6**	10.3	14.8	**9.5**	7.5	11.4	**0.3**	0.2	1.2	**0.2**	0.1	0.2
**2637.004**	R3	**33.9**	28.5	39.3	25.0 *	18.6	31.3	20.6 *	14.9	26.2	10.1 *	6.5	13.7	**4.8**	1.0	8.6
**2637.019**	R3	**31.9**	24.6	39.2	33.1 *	25.6	40.5	**11.5**	9.3	13.7	**0.3**	0.3	0.3	**NA**	NA	NA
**2637.007**	R5	**29.9**	25.2	34.6	**14.8**	10.3	19.3	**8.8**	6.9	10.7	4.0 *	2.4	5.6	**3.0**	0.8	5.2
**2637.010**	R5	**31.0**	25.6	36.4	**9.2**	7.6	10.8	**5.5**	4.1	6.9	**1.7**	0.3	3.3	**1.0**	0.2	1.8
**2637.012**	R5	**33.3**	27.4	39.1	25.9 *	21.8	29.9	20.6 *	17.9	23.3	10.4 *	9.1	11.6	**0.3**	0.3	0.3
**2637.011**	R5	**22.7**	18.3	27.0	**16.0**	14.0	18.0	**11.0**	9.9	12.2	**0.6**	0.2	1.9	**0.3**	0.3	0.3
**2637.013**	R5	**29.7**	24.2	35.2	**15.3**	12.6	18.1	**8.0**	5.9	10.0	**0.7**	0.2	1.7	**0.1**	0.2	0.5
**2637.014**	R5	**33.6**	29.1	38.1	24.9 *	20.4	29.4	19.5 *	14.8	24.2	9.1 *	3.6	14.6	**2.8**	0.6	4.9

IC_50_ with 95% confidence intervals for compounds tested in the checkerboard assays. All values are based on curve-fitting of Hill’s equation using least squares regression. These curves are shown in Figure S7. * indicates a non-overlapping confidence interval demonstrating a significant reduction in the potentiation ability of amikacin at that dose. Underlining indicates IC_50_ values less than half of the minimal checkerboard dose, thus being interpolate estimates. NA, not applicable.

## Data Availability

All data described in the study can be found in the article or in the supplementary materials.

## Supplementary Materials

Supplementary information is available online at https://www.mdpi.com/article/10.3390/biomedicines9091218/s1, Figure S1: Compound synthesis, Figure S2: Compound properties and purity, Figure S3: Representative graphics corresponding to the analysis and purity determination of compounds (shown for compounds **2637.001** and **2637.002**), Figure S4: Growth curve of A. baumannii A155 cultured in liquid medium with no additions, addition of 16 μg/mL amikacin, and addition of 16 μg/mL amikacin plus 8 mM compound **2637.001**, Figure S5: Complex between AAC(6′)-Ib and amikacin, Figure S6: Checkerboard assays, Figure S7: Fractional inhibition curves for all compounds tested in the checkerboard assays.
