# Supplementary material for: Inhibition of Aminoglycoside 6′-N-acetyltransferase Type Ib (AAC(6′)-Ib): Structure–Activity Relationship of Substituted Pyrrolidine Pentamine Derivatives as Inhibitors
Source: Biomedicines. 2021 Sep 14;9(9):1218. doi: 10.3390/biomedicines9091218 (PMC8471502; doi:10.3390/biomedicines9091218)
Supplement: Supplementary file 1 [file biomedicines-09-01218-s001.zip › biomedicines-1359714-supplementary.pdf]

Supplementary Materials

Inhibition of aminoglycoside 6'-*N*-acetyltransferase type Ib [AAC(6′)-Ib]: structure-activity relationship of substituted pyrrolidine pentamine derivatives as inhibitors

Kenneth Rocha[1], Jesus Magallon[1], Craig Reeves[1], Kimberly Phan[1], Peter Vu[1], Crista L. Oakley-Havens[1], Stella Kwan[1], Maria S. Ramirez[1], Travis LaVoi[2], Haley Donow[2], Prem Chapagain[3,4], Radleigh Santos[5], Clemencia Pinilla[2], Marc A. Giulianotti[2], and Marcelo E. Tolmasky[1]*

[1] Center for Applied Biotechnology Studies, Department of Biological Science, College of Natural Sciences and Mathematics, California State University Fullerton, Fullerton, CA

[2] Center for Translational Science, Florida International University, Port St. Lucie, FL

[3] Department of Physics, Florida International University, Miami, FL

[4] Biomolecular Sciences Institute, Florida International University, Miami, FL

[5] Nova Southeastern University, Fort Lauderdale, FL

* Correspondence: mtolmasky@fullerton.edu; Tel.: 1-657-278-5263

Figure S1

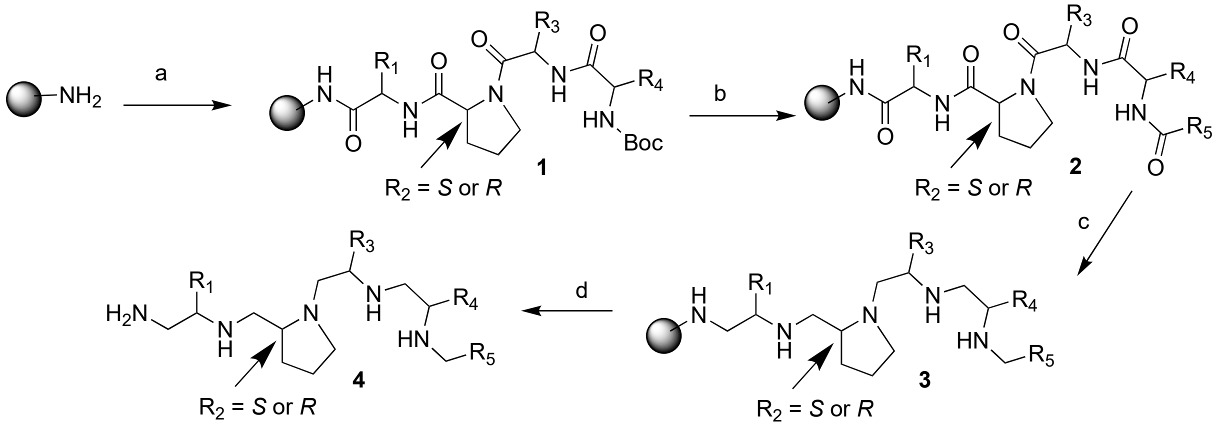

Figure S1. a) standard Boc coupling protocol utilizing repetitive a1) 5% DIEA / 95% DCM (vol%); a2) Boc-AA, DIC, HOBt, DMF; a3) 55% TFA / 45% DCM (vol%); **1**. b) 5% DIEA / 95% DCM (vol%); R^4COOH, DIC, HOBt, DMF; **2**. c) 40 x BH3 / THF (65 °C, 72 hr); piperidine (65 °C, 18 hr); **3**. d) HF / Anisole, (0 °C, 7 hr).

Figure S2

Chemical characterization of compounds listed in Table 1

Compound **2637.001**, (2S)-3-((S)-2-(((S)-2-amino-1-phenylethylamino)methyl)pyrrolidin-1-yl)- 2-((2S)-2-phenyl-2-(3-phenylbutylamino)ethylamino)propan-1-ol, Using General Scheme (SI Figure 3) for the synthesis of penta-amines compound 2637.001 was synthesized using the following reagents: (R1) Boc-L-Phenyglycine-OH, (R2) Boc-D-Serine(Bzl)-OH, (R3) Boc-L-Phenyglycine-OH, (R4) 3-Phenylbutyric Acid. **m/z** calcd $C_{34}H_{49}N_5O$ $[M+H]^+$ 543.79, found (MS ESI) 544.3. **Purity** LCMS: 100% (TIC), 100% (254 nm, peak area); **RT** = 6.991 min.

Compound **2637.002**, (2S)-3-((S)-2-((((S)-1-aminopropan-2-yl)amino)methyl)pyrrolidin-1-yl)-2-(((2S)-2-phenyl-2-((3-phenylbutyl)amino)ethyl)amino)propan-1-ol, Using General Scheme (SI Figure 3) for the synthesis of penta-amines compound 2637.001 was synthesized using the following reagents: (R1) Boc-L-Alanine, (R2) Boc-D-Serine(Bzl)-OH, (R3) Boc-L-Phenyglycine-OH, (R4) 3-Phenylbutyric Acid. **m/z** calcd $C_{29}H_{47}N_5O$ $[M+H]^+$ 481.72, found (MS ESI) 482.2. **Purity** LCMS: 100% (TIC), 100% (254 nm, peak area); **RT** = 6.376 min.

Compound **2637.003**, (2S)-3-((S)-2-((((S)-1-amino-3-phenylpropan-2-yl)amino)methyl)pyrrolidin-1-yl)-2-(((2S)-2-phenyl-2-((3-phenylbutyl)amino)ethyl)amino)propan-1-ol, Using General Scheme (SI Figure 3) for the synthesis of penta-amines compound 2637.001 was synthesized using the following reagents: (R1) Boc-L-Phenylalanine, (R2) Boc-D-Serine(Bzl)-OH, (R3) Boc-L-Phenyglycine-OH, (R4) 3-Phenylbutyric Acid. **m/z** calcd $C_{35}H_{51}N_5O$ $[M+H]^+$ 557.81, found (MS ESI) 558.2. **Purity** LCMS: 99.1% (TIC), 100% (254 nm, peak area); **RT** = 3.413 min.

Compound **2637.004**, (1R)-N2-((S)-1-((S)-2-((((S)-2-amino-1-phenylethyl)amino)methyl)pyrrolidin-1-yl)propan-2-yl)-1-phenyl-N1-(3-phenylbutyl)ethane-1,2-diamine, Using General Scheme (SI Figure 3) for the synthesis of penta-amines compound 2637.001 was synthesized using the following reagents: (R1) Boc-L-Phenyglycine-OH, (R2) Boc-L-Alanine-OH, (R3) Boc-L-Phenyglycine-OH, (R4) 3-Phenylbutyric Acid. **m/z** calcd $C_{34}H_{49}N_5$ $[M+H]^+$ 527.79, found (MS ESI) 528.2. **Purity** LCMS: 100% (TIC), 100% (254 nm, peak area); **RT** = 7.136 min.

Compound **2637.005**, (2R)-3-((S)-2-((((S)-2-amino-1-phenylethyl)amino)methyl)pyrrolidin-1-yl)-2-(((2S)-2-phenyl-2-((3-phenylbutyl)amino)ethyl)amino)propan-1-ol, Using General Scheme (SI Figure 3) for the synthesis of penta-amines compound 2637.001 was synthesized using the following reagents: (R1) Boc-L-Phenyglycine-OH, (R2) Boc-L-Serine(Bzl)-OH, (R3) Boc-L-Phenyglycine-OH, (R4) 3-Phenylbutyric Acid. **m/z** calcd $C_{34}H_{49}N_5O$ $[M+H]^+$ 543.79, found (MS ESI) 544.3. **Purity** LCMS: 100% (TIC), 100% (254 nm, peak area); **RT** = 7.103 min.

Compound **2637.006**, (2S)-3-((S)-2-((((S)-2-amino-1-phenylethyl)amino)methyl)pyrrolidin-1-yl)-2-(((2S)-2-((3-phenylbutyl)amino)propyl)amino)propan-1-ol, Using General Scheme (SI Figure 3) for the synthesis of penta-amines compound 2637.001 was synthesized using the following reagents: (R1) Boc-L-Phenyglycine-OH, (R2) Boc-D-Serine(Bzl)-OH, (R3) Boc-L-Alanine-OH, (R4) 3-Phenylbutyric Acid. **m/z** calcd $C_{29}H_{47}N_5O$ $[M+H]^+$ 481.72, found (MS ESI) 482.2 **Purity** LCMS: 100% (TIC), 84.6% (254 nm, peak area); **RT** = 6.138 min.

Figure S2 continued

Compound **2637.007**, (S)-3-((S)-2-((((S)-2-amino-1-phenylethyl)amino)methyl)pyrrolidin-1-yl)-2-(((S)-2-(ethylamino)-2-phenylethyl)amino)propan-1-ol, Using General Scheme (SI Figure 3) for the synthesis of penta-amines compound 2637.001 was synthesized using the following reagents: (R1) Boc-L-Phenyglycine-OH, (R2) Boc-D-Serine(Bzl)-OH, (R3) Boc-L-Phenyglycine-OH, (R4) Acetic acid. **m/z** calcd $C_{26}H_{41}N_5O$ $[M+H]^+$ 439.64, found (MS ESI) 440.2. **Purity** LCMS: 100% (TIC), 100% (254 nm, peak area); **RT** = 1.231 min.

Compound **2637.008**, (S)-3-((S)-2-((((S)-2-amino-1-phenylethyl)amino)methyl)pyrrolidin-1-yl)-2-(((S)-2-(pentylamino)-2-phenylethyl)amino)propan-1-ol, Using General Scheme (SI Figure 3) for the synthesis of penta-amines compound 2637.001 was synthesized using the following reagents: (R1) Boc-L-Phenyglycine-OH, (R2) Boc-D-Serine(Bzl)-OH, (R3) Boc-L-Phenyglycine-OH, (R4) Butyric Acid. **m/z** calcd $C_{29}H_{47}N_5O$ $[M+H]^+$ 481.72, found (MS ESI) 482.15. **Purity** LCMS: 100% (TIC), 95.4% (254 nm, peak area); **RT** = 3.246 min.

Compound **2637.009**, (S)-3-((S)-2-((((S)-2-amino-1-phenylethyl)amino)methyl)pyrrolidin-1-yl)-2-(((S)-2-phenyl-2-((2-phenylbutyl)amino)ethyl)amino)propan-1-ol, Using General Scheme (SI Figure 3) for the synthesis of penta-amines compound 2637.001 was synthesized using the following reagents: (R1) Boc-L-Phenyglycine-OH, (R2) Boc-D-Serine(Bzl)-OH, (R3) Boc-L-Phenyglycine-OH, (R4) 2-Phenylbutyric Acid. **m/z** calcd $C_{34}H_{49}N_5O$ $[M+H]^+$ 543.79, found (MS ESI) 544.2. **Purity** LCMS: 99.4% (TIC), 100% (254 nm, peak area); **RT** = 3.208 min.

Compound **2637.010**, (S)-3-((S)-2-((((S)-2-amino-1-phenylethyl)amino)methyl)pyrrolidin-1-yl)-2-(((S)-2-(pentylamino)-2-phenylethyl)amino)propan-1-ol, Using General Scheme (SI Figure 3) for the synthesis of penta-amines compound 2637.001 was synthesized using the following reagents: (R1) Boc-L-Phenyglycine-OH, (R2) Boc-D-Serine(Bzl)-OH, (R3) Boc-L-Phenyglycine-OH, (R4) Valeric Acid. **m/z** calcd $C_{29}H_{47}N_5O$ $[M+H]^+$ 481.72, found (MS ESI) 482.15. **Purity** LCMS: 100% (TIC), 100% (254 nm, peak area); **RT** = 2.700 min.

Compound **2637.011**, (S)-3-((S)-2-((((S)-2-amino-1-phenylethyl)amino)methyl)pyrrolidin-1-yl)-2-(((S)-2-phenyl-2-((4-phenylbutyl)amino)ethyl)amino)propan-1-ol, Using General Scheme (SI Figure 3) for the synthesis of penta-amines compound 2637.001 was synthesized using the following reagents: (R1) Boc-L-Phenyglycine-OH, (R2) Boc-D-Serine(Bzl)-OH, (R3) Boc-L-Phenyglycine-OH, (R4) 4-Phenylbutyric Acid. **m/z** calcd $C_{34}H_{49}N_5O$ $[M+H]^+$ 543.79, found (MS ESI) 544.2. **Purity** LCMS: 99.6% (TIC), 100% (254 nm, peak area); **RT** = 3.237 min.

Compound **2637.012**, (S)-3-((S)-2-((((S)-2-amino-1-phenylethyl)amino)methyl)pyrrolidin-1-yl)-2-(((S)-2-phenyl-2-((2-phenylbutyl)amino)ethyl)amino)propan-1-ol, Using General Scheme (SI Figure 3) for the synthesis of penta-amines compound 2637.001 was synthesized using the following reagents: (R1) Boc-L-Phenyglycine-OH, (R2) Boc-D-Serine(Bzl)-OH, (R3) Boc-L-Phenyglycine-OH, (R4) 2-Phenylbutyric Acid. **m/z** calcd $C_{34}H_{49}N_5O$ $[M+H]^+$ 543.79, found (MS ESI) 544.2. **Purity** LCMS: 99.4% (TIC), 100% (254 nm, peak area); **RT** = 3.191 min.

Figure S2 continued

Compound **2637.013**, (S)-3-((S)-2-((((S)-2-amino-1-phenylethyl)amino)methyl)pyrrolidin-1-yl)-2-(((S)-2-phenyl-2-((3-phenylpropyl)amino)ethyl)amino)propan-1-ol, Using General Scheme (SI Figure 3) for the synthesis of penta-amines compound 2637.001 was synthesized using the following reagents: (R1) Boc-L-Phenyglycine-OH, (R2) Boc-D-Serine(Bzl)-OH, (R3) Boc-L-Phenyglycine-OH, (R4) 3-Phenylpropanoic acid. **m/z** calcd $C_{33}H_{47}N_5O$ $[M+H]^+$ 529.76, found (MS ESI) 530.2. **Purity** LCMS: 99.1% (TIC),100% (254 nm, peak area); **RT** = 3.041 min.

Compound **2637.014**, (S)-3-((S)-2-((((S)-2-amino-1-phenylethyl)amino)methyl)pyrrolidin-1-yl)-2-(((S)-2-phenyl-2-((3-(pyridin-3-yl)propyl)amino)ethyl)amino)propan-1-ol, Using General Scheme (SI Figure 3) for the synthesis of penta-amines compound 2637.001 was synthesized using the following reagents: (R1) Boc-L-Phenyglycine-OH, (R2) Boc-D-Serine(Bzl)-OH, (R3) Boc-L-Phenyglycine-OH, (R4) 3-pyridionepropionic acid. **m/z** calcd $C_{32}H_{46}N_6O$ $[M+H]^+$ 530.75, found (MS ESI) 531.2. **Purity** LCMS: 99.5% (TIC), 100% (254 nm, peak area); **RT** = 1.087 min.

Compound **2637.015**, (S)-3-((S)-2-((((S)-2-amino-1-phenylethyl)amino)methyl)pyrrolidin-1-yl)-2-(((S)-2-amino-2-phenylethyl)amino)propan-1-ol, Using General Scheme (SI Figure 3) for the synthesis of penta-amines compound 2637.001 was synthesized using the following reagents: (R1) Boc-L-Phenyglycine-OH, (R2) Boc-D-Serine(Bzl)-OH, (R3) Boc-L-Phenyglycine-OH. **m/z** calcd $C_{24}H_{37}N_5O$ $[M+H]^+$ 411.58, found (MS ESI) 412.2. **Purity** LCMS: 99.0% (TIC), 100% (254 nm, peak area); **RT** = 1.072 min.

Compound **2637.016**, (S)-3-((S)-2-((((S)-2-amino-1-phenylethyl)amino)methyl)pyrrolidin-1-yl)-2-(((S)-2-aminopropyl)amino)propan-1-ol, Using General Scheme (SI Figure 3) for the synthesis of penta-amines compound 2637.001 was synthesized using the following reagents: (R1) Boc-L-Phenyglycine-OH, (R2) Boc-D-Serine(Bzl)-OH, (R3) Boc-L-Alanine-OH. **m/z** calcd $C_{19}H_{35}N_5O$ $[M+H]^+$ 349.51, found (MS ESI) 350.2. **Purity** LCMS: 100% (TIC), 100% (254 nm, peak area); **RT** = 1.009 min.

Compound **2637.017**, (S)-3-((S)-2-((((S)-2-amino-1-phenylethyl)amino)methyl)pyrrolidin-1-yl)-2-(ethylamino)propan-1-ol, Using General Scheme (SI Figure 3) for the synthesis of penta-amines compound 2637.001 was synthesized using the following reagents: (R1) Boc-L-Phenyglycine-OH, (R2) Boc-D-Serine(Bzl)-OH, (R3) Acetic acid. **m/z** calcd $C_{18}H_{32}N_4O$ $[M+H]^+$ 320.47, found (MS ESI) 321.2. **Purity** LCMS: 100% (TIC), 100% (254 nm, peak area); **RT** = 1.034 min.

Compound **2637.018**, (S)-2-amino-3-((S)-2-((((S)-2-amino-1-phenylethyl)amino)methyl)pyrrolidin-1-yl)propan-1-ol, Using General Scheme (SI Figure 3) for the synthesis of penta-amines compound 2637.001 was synthesized using the following reagents: (R1) Boc-L-Phenyglycine-OH, (R2) Boc-D-Serine(Bzl)-OH. **m/z** calcd $C_{16}H_{28}N_4O$ $[M+H]^+$ 292.42, found (MS ESI) 293.1. **Purity** LCMS: 100% (TIC), 100% (254 nm, peak area); **RT** = 1.026 min.

Compound **2637.019**, (1S)-N2-((R)-1-((S)-2-((((S)-2-amino-1-phenylethyl)amino)methyl)pyrrolidin-1-yl)propan-2-yl)-1-phenyl-N1-(3-phenylbutyl)ethane-1,2-diamine, Using General Scheme (SI Figure 3) for the synthesis of penta-amines compound 2637.001 was synthesized using the following reagents: (R1) Boc-L-Phenyglycine-OH, (R2) Boc-D-Alanine-OH, (R3) Boc-L-Phenyglycine-OH, (R4) 3-Phenylbutyric Acid. **m/z** calcd $C_{34}H_{49}N_5$ $[M+H]^+$ 527.79, found (MS ESI) 528.2. **Purity** LCMS: 96.3% (TIC), 100% (254 nm, peak area); **RT** = 3.263 min.

Figure S2 continued

Compound **2637.020**, (2S)-3-((S)-2-((((R)-2-amino-1-phenylethyl)amino)methyl)pyrrolidin-1-yl)-2-(((2S)-2-phenyl-2-((3-phenylbutyl)amino)ethyl)amino)propan-1-ol, Using General Scheme (SI Figure 3) for the synthesis of penta-amines compound 2637.001 was synthesized using the following reagents: (R1) Boc-D-Phenyglycine-OH, (R2) Boc-D-Serine(Bzl)-OH, (R3) Boc-L-Phenyglycine-OH, (R4) 3-Phenylbutyric Acid. **m/z** calcd $C_{34}H_{49}N_5O$ $[M+H]^+$ 543.79, found (MS ESI) 544.2. **Purity** LCMS: 99.1% (TIC), 100% (254 nm, peak area); **RT** = 3.2 04min.

Compound **2637.021**, (2S)-3-((R)-2-((((R)-2-amino-1-phenylethyl)amino)methyl)pyrrolidin-1-yl)-2-(((2S)-2-phenyl-2-((3-phenylbutyl)amino)ethyl)amino)propan-1-ol, Using General Scheme (SI Figure 3) for the synthesis of penta-amines compound 2637.001 was synthesized using the following reagents: (R1) Boc-L-Phenyglycine-OH, (R2) Boc-D-Serine(Bzl)-OH, (R3) Boc-L-Phenyglycine-OH, (R4) 3-Phenylbutyric Acid. **m/z** calcd $C_{34}H_{49}N_5O$ $[M+H]^+$ 543.79, found (MS ESI) 544.2. **Purity** LCMS: 99.3% (TIC), 100% (254 nm, peak area); **RT** = 3.225min.

Compound **2637.022**, (2S)-3-((S)-2-((((R)-2-amino-1-phenylethyl)amino)methyl)pyrrolidin-1-yl)-2-(((2R)-2-phenyl-2-((3-phenylbutyl)amino)ethyl)amino)propan-1-ol, Using General Scheme (SI Figure 3) for the synthesis of penta-amines compound 2637.001 was synthesized using the following reagents: (R1) Boc-L-Phenyglycine-OH, (R2) Boc-D-Serine(Bzl)-OH, (R3) Boc-L-Phenyglycine-OH, (R4) 3-Phenylbutyric Acid. **m/z** calcd $C_{34}H_{49}N_5O$ $[M+H]^+$ 543.79, found (MS ESI) 544.2. **Purity** LCMS: 98.9% (TIC), 100% (254 nm, peak area); **RT** = 3.229 min.

Figure S2. The compounds listed in Table 1 were synthesized using the conditions described in Figure S1. The desired product was cleaved from the solid support resin and extracted using 95% acetic acid. Samples were then repeatedly frozen and lyophilized in 50% acetonitrile and water. Confirmation of the desired product was obtained by reverse phase LCMS analysis utilizing the same method as described in the "LCMS Analysis of Purified Material" section. During purification the peak corresponding to the desired product with calculated m/z was isolated and concentrated.

Figure S3

2637.001

UV @ 254nm

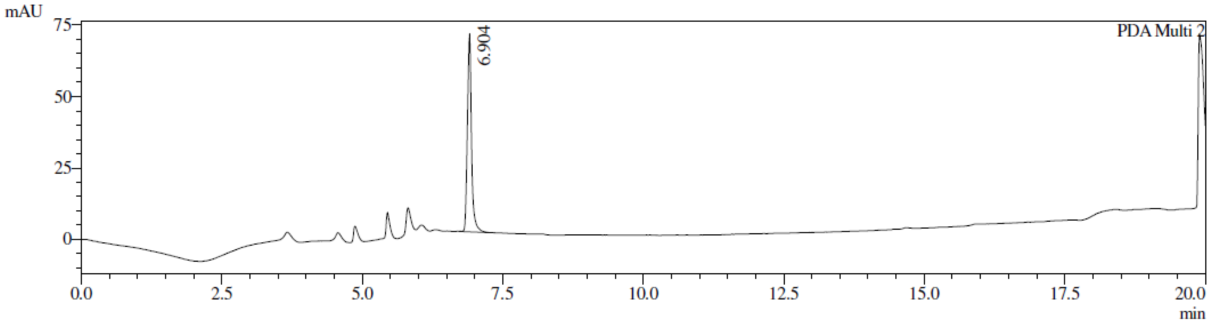

PeakTable

PDA Ch2 254nm 4nm

Peak#	Ret. Time	Area	Area %
1	6.904	348544	100.000
Total		348544	100.000

MS

MS Chromatogram
2637-1

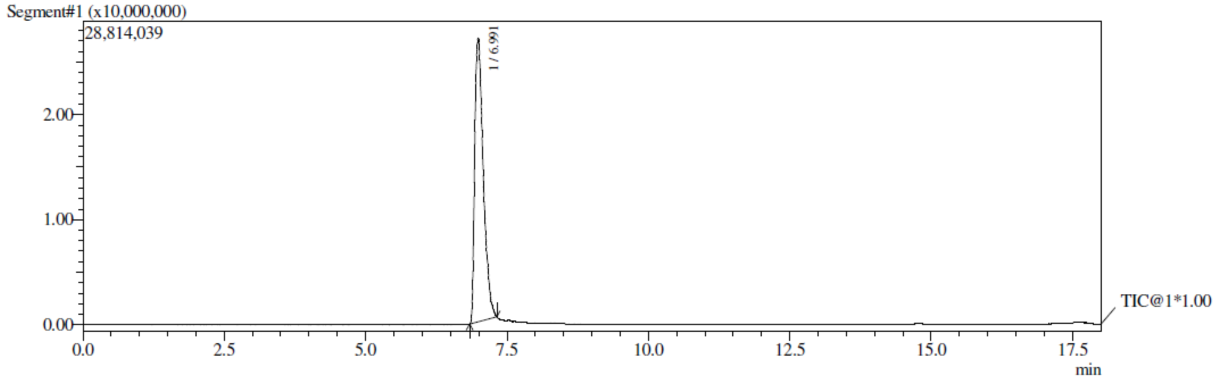

MS Spectrum Graph

Peak#:1 Ret.Time:Averaged 6.987-6.993(Scan#:2097-2099)
BG Mode:Calc 6.840<->7.320(2053<->2197)
Mass Peaks:70 Base Peak:272.65(12012280) Polarity:Pos Segment1 - Event1

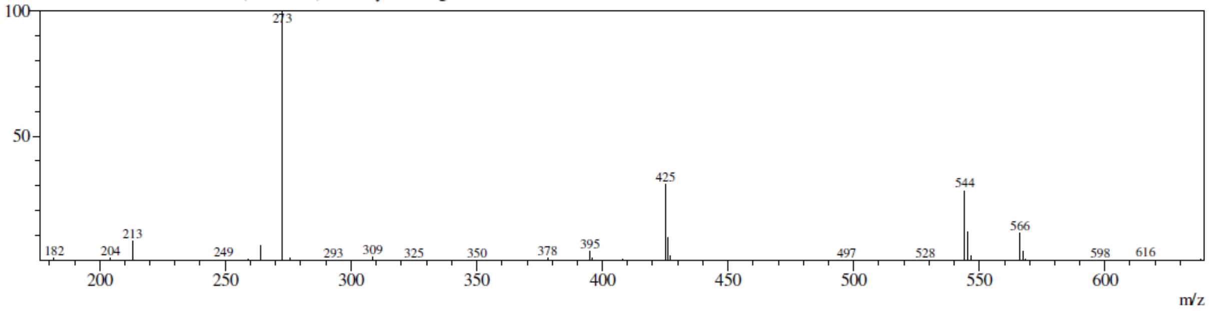

MS Peak Table TIC

Peak#	Ret.Time	Peak Start	Peak End	Area	Area%	Height	Height%	A/H	Mark	Name	ID#	Event#
1	6.991	6.840	7.320	284894567	100.00	26981224	100.00	10.55				1-1
**Total**				284894567	100.00	26981224	100.00					

Figure S3 continued

2637.002

UV @ 254nm

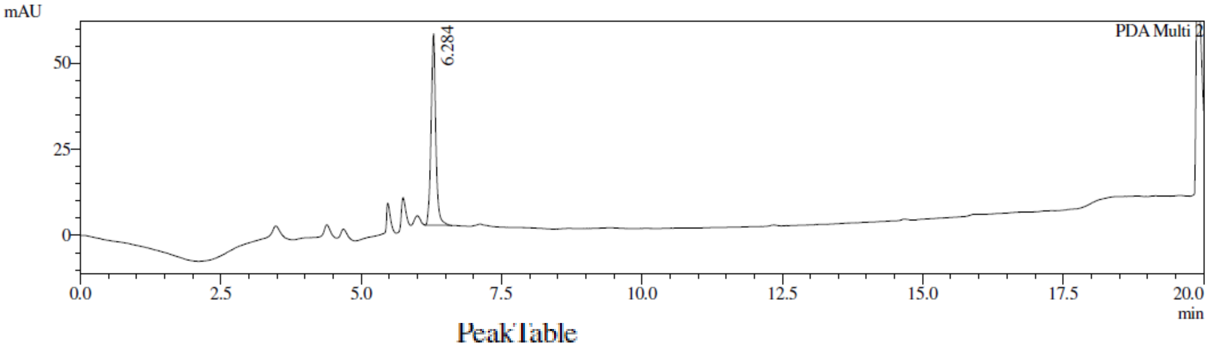

PeakTable

PDA Ch2 254nm 4nm

Peak#	Ret. Time	Area	Area %
1	6.284	320256	100.000
Total		320256	100.000

MS

MS Chromatogram
2637-2 with AA

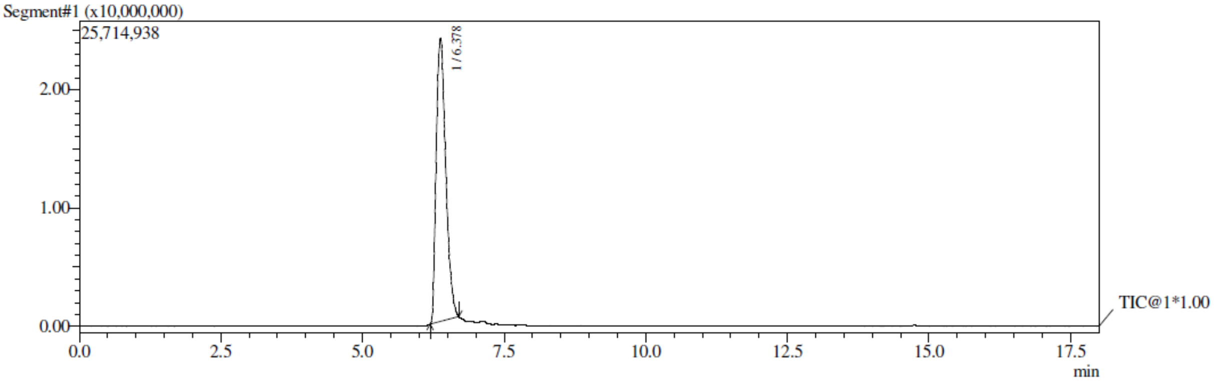

MS Spectrum Graph

Peak#:1 Ret.Time:Averaged 6.373-6.380(Scan#:1913-1915)
BG Mode:Calc 6.193<->6.700(1859<->2011)
Mass Peaks:64 Base Peak:241.65(13772507) Polarity:Pos Segment1 - Event1

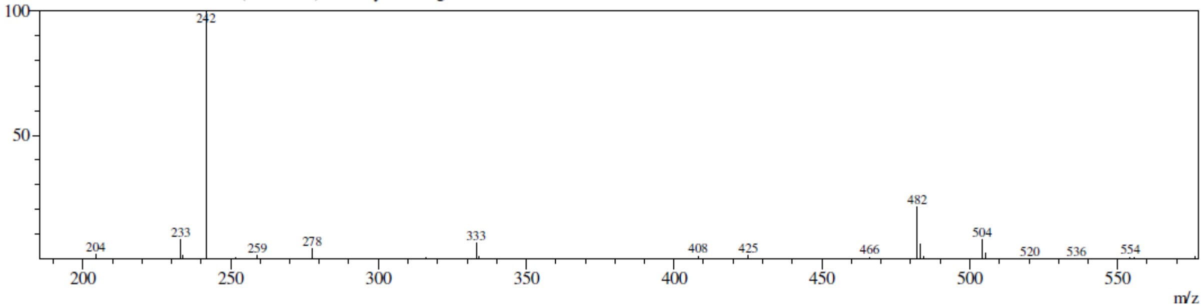

MS Peak Table TIC

Peak#	Ret.Time	Peak Start	Peak End	Area	Area%	Height	Height%	A/H	Mark	Name	ID#	Event#
1	6.378	6.193	6.700	280192889	100.00	23924900	100.00	11.71				1-1
**Total**				280192889	100.00	23924900	100.00					

Figure S3. LCMS data for compounds 2637.001 and 2637.002. Shown for each is the UV trace at 254 nM, the % purity integration data, the Total Ion Current (TIC) spectra, the Mass Spectrum (MS) for the product peak, and the MS peak table.

Figure S4

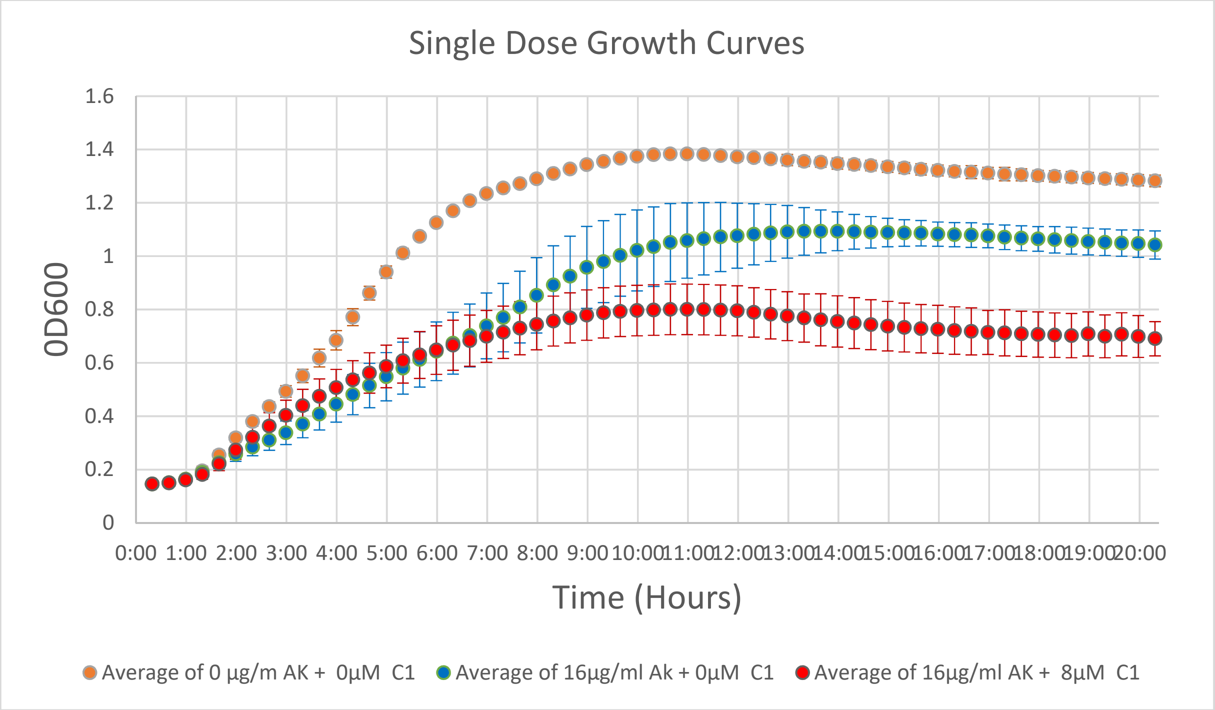

Figure S4. Growth curve of *A. baumannii* A155 cultured in Mueller Hinton broth without additions, with the addition of 16 µg/mL amikacin (Ak), and 16 µg/mL amikacin plus 8 µM compound 2637.001 (C1). The experiment was carried five times by duplicate.

Figure S5

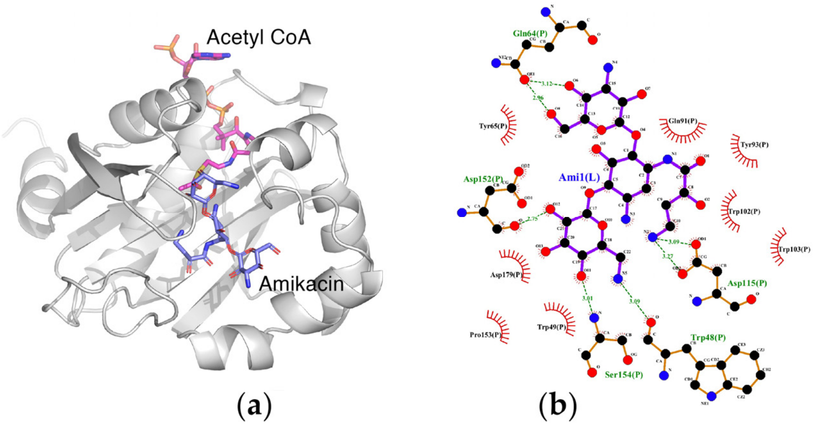

Figure S5. (a) The complex of amikacin and AAC(6′)-Ib obtained from molecular docking. The bound acetyl CoA is also shown. **(b)** Amikacin has hydrogen bonds with side chain of the charged residues Glu64, Asp115, and Asp52 as well as hydrogen bonds with the backbone of resides Ser154 and Trp48.

Figure S6

Adjusted values (antimicrobial activity of compounds removed)

	Amk (µg/mL)					
2637-001 (µM)	0	8	16	24	32	64
24.0	10.8%	84.8%	88.2%	89.7%	88.5%	88.3%
16.0	4.3%	78.7%	85.1%	87.9%	88.9%	88.9%
8.0	8.6%	50.2%	74.5%	84.2%	87.4%	87.0%
4.0	6.7%	33.2%	63.2%	77.2%	85.0%	85.9%
0.0	0.0%	8.9%	29.4%	27.4%	52.8%	89.4%

	Amk (µg/mL)					
2637-001 (µM)	0	8	16	24	32	64
24.0	0.0%	83.0%	86.8%	88.4%	87.1%	86.9%
16.0	0.0%	77.7%	84.5%	87.3%	88.4%	88.4%
8.0	0.0%	45.6%	72.1%	82.8%	86.2%	85.8%
4.0	0.0%	28.4%	60.6%	75.5%	84.0%	84.9%
0.0	0.0%	8.9%	29.4%	27.4%	52.8%	89.4%

	Amk (µg/mL)					
2637.020 (µM)	0	8	16	24	32	64
24.0	6.3%	86.6%	87.2%	77.4%	89.7%	91.8%
16.0	6.3%	82.6%	88.0%	88.1%	87.8%	90.9%
8.0	10.2%	43.9%	79.4%	87.2%	88.8%	88.5%
4.0	8.2%	34.5%	58.0%	85.3%	79.8%	90.9%
0.0	0.0%	8.0%	23.7%	41.4%	75.4%	91.5%

	Amk (µg/mL)					
2637.020 (µM)	0	8	16	24	32	64
24.0	0.0%	85.7%	86.3%	75.9%	88.9%	91.2%
16.0	0.0%	81.5%	87.2%	87.3%	86.9%	90.3%
8.0	0.0%	37.5%	77.0%	85.7%	87.5%	87.2%
4.0	0.0%	28.7%	54.3%	84.0%	78.0%	90.1%
0.0	0.0%	8.0%	23.7%	41.4%	75.4%	91.5%

	Amk (µg/mL)					
2637.004 (µM)	0	8	16	24	32	64
24.0	5.0%	88.4%	89.6%	90.5%	90.7%	90.7%
16.0	3.5%	25.5%	85.4%	88.4%	85.3%	86.9%
8.0	5.3%	14.7%	47.1%	52.7%	77.1%	87.8%
4.0	6.0%	13.7%	40.9%	45.6%	55.8%	86.3%
0.0	0.0%	6.8%	22.0%	20.4%	27.7%	88.2%

	Amk (µg/mL)					
2637.004 (µM)	0	8	16	24	32	64
24.0	0.0%	87.8%	89.1%	90.0%	90.2%	90.2%
16.0	0.0%	22.9%	84.9%	88.0%	84.8%	86.4%
8.0	0.0%	9.9%	44.1%	50.0%	75.8%	87.2%
4.0	0.0%	8.3%	37.2%	42.2%	53.0%	85.4%
0.0	0.0%	6.8%	22.0%	20.4%	27.7%	88.2%

	Amk (µg/mL)					
2637.019 (µM)	0	8	16	24	32	64
24.0	90.8%	91.2%	90.8%	91.0%	91.1%	91.3%
16.0	19.1%	91.3%	90.5%	89.8%	90.6%	91.0%
8.0	5.9%	26.1%	87.8%	81.7%	89.1%	90.3%
4.0	8.0%	16.1%	30.2%	36.0%	26.1%	90.5%
0.0	0.0%	7.2%	16.2%	26.6%	23.8%	90.7%

	Amk (µg/mL)					
2637.019 (µM)	0	8	16	24	32	64
24.0	0.0%	4.9%	0.7%	2.4%	3.9%	5.5%
16.0	0.0%	89.2%	88.3%	87.4%	88.4%	88.9%
8.0	0.0%	21.5%	87.0%	80.6%	88.4%	89.7%
4.0	0.0%	8.8%	24.1%	30.5%	19.7%	89.7%
0.0	0.0%	7.2%	16.2%	26.6%	23.8%	90.7%

	Amk (µg/mL)					
2637.007 (µM)	0	8	16	24	32	64
24.0	1.6%	63.7%	67.8%	79.0%	77.6%	82.2%
16.0	1.3%	61.0%	72.6%	78.3%	78.8%	81.1%
8.0	2.2%	51.4%	70.5%	79.3%	75.7%	82.3%
4.0	3.6%	39.4%	61.9%	78.1%	52.5%	88.7%
0.0	0.0%	6.9%	16.0%	26.4%	42.6%	89.1%

	Amk (µg/mL)					
2637.007 (µM)	0	8	16	24	32	64
24.0	0.0%	63.2%	67.3%	78.6%	77.3%	81.9%
16.0	0.0%	60.5%	72.3%	78.0%	78.5%	80.8%
8.0	0.0%	50.3%	69.8%	78.8%	75.2%	81.9%
4.0	0.0%	37.1%	60.5%	77.3%	50.7%	88.3%
0.0	0.0%	6.9%	16.0%	26.4%	42.6%	89.1%

	Amk (µg/mL)					
2637.010 (µM)	0	8	16	24	32	64
24.0	4.6%	71.8%	78.8%	81.4%	81.1%	87.2%
16.0	4.5%	68.8%	80.0%	78.7%	81.6%	86.2%
8.0	4.8%	60.5%	71.8%	79.8%	84.4%	87.9%
4.0	4.3%	50.7%	69.6%	78.7%	83.6%	87.6%
0.0	0.0%	4.6%	25.5%	20.3%	65.5%	89.1%

	Amk (µg/mL)					
2637.010 (µM)	0	8	16	24	32	64
24.0	0.0%	70.4%	77.8%	80.5%	80.2%	86.6%
16.0	0.0%	67.3%	79.1%	77.7%	80.7%	85.5%
8.0	0.0%	58.5%	70.4%	78.8%	83.7%	87.3%
4.0	0.0%	48.5%	68.2%	77.8%	82.9%	87.1%
0.0	0.0%	4.6%	25.5%	20.3%	65.5%	89.1%

	Amk (µg/mL)					
2637.012 (µM)	0	8	16	24	32	64
24.0	-0.7%	89.5%	89.4%	87.6%	85.1%	91.5%
16.0	0.5%	33.5%	79.2%	83.3%	85.9%	87.9%
8.0	2.7%	15.6%	44.0%	54.0%	70.2%	88.3%
4.0	4.8%	12.2%	33.4%	57.9%	54.4%	85.7%
0.0	0.0%	8.9%	16.8%	21.8%	52.1%	86.0%

	Amk (µg/mL)					
2637.012 (µM)	0	8	16	24	32	64
24.0	0.0%	89.6%	89.5%	87.7%	85.2%	91.6%
16.0	0.0%	33.2%	79.0%	83.2%	85.8%	87.9%
8.0	0.0%	13.3%	42.4%	52.7%	69.4%	88.0%
4.0	0.0%	7.8%	30.0%	55.7%	52.1%	85.0%
0.0	0.0%	8.9%	16.8%	21.8%	52.1%	86.0%

	Amk (µg/mL)					
2637.011 (µM)	0	8	16	24	32	64
24.0	2.5%	86.1%	83.6%	80.8%	83.1%	82.0%
16.0	1.8%	77.6%	85.4%	86.2%	86.7%	88.6%
8.0	4.7%	36.7%	70.4%	80.6%	84.8%	87.0%
4.0	4.8%	27.5%	46.5%	69.3%	82.1%	86.2%
0.0	0.0%	3.7%	31.3%	54.5%	76.2%	87.1%

	Amk (µg/mL)					
2637.011 (µM)	0	8	16	24	32	64
24.0	0.0%	85.7%	83.2%	80.3%	82.6%	81.5%
16.0	0.0%	77.2%	85.2%	85.9%	86.4%	88.4%
8.0	0.0%	33.5%	68.9%	79.6%	84.0%	86.4%
4.0	0.0%	23.9%	43.8%	67.8%	81.2%	85.5%
0.0	0.0%	3.7%	31.3%	54.5%	76.2%	87.1%

	Amk (µg/mL)					
2637.013 (µM)	0	8	16	24	32	64
24.0	2.4%	81.2%	83.7%	88.3%	83.3%	90.0%
16.0	3.9%	74.3%	84.1%	87.5%	86.2%	86.0%
8.0	5.0%	48.2%	76.1%	85.2%	78.4%	88.4%
4.0	4.9%	25.2%	55.9%	77.5%	58.4%	87.5%
0.0	0.0%	5.6%	33.1%	31.2%	57.2%	89.0%

	Amk (µg/mL)					
2637.013 (µM)	0	8	16	24	32	64
24.0	0.0%	80.7%	83.3%	88.0%	82.9%	89.7%
16.0	0.0%	73.2%	83.5%	87.0%	85.7%	85.4%
8.0	0.0%	45.5%	74.8%	84.4%	77.3%	87.8%
4.0	0.0%	21.3%	53.6%	76.3%	56.3%	86.9%
0.0	0.0%	5.6%	33.1%	31.2%	57.2%	89.0%

	Amk (µg/mL)					
2637.014 (µM)	0	8	16	24	32	64
24.0	3.9%	61.1%	73.1%	75.2%	77.7%	78.0%
16.0	3.3%	57.0%	65.2%	64.1%	57.8%	87.5%
8.0	2.9%	41.4%	43.6%	52.8%	54.3%	84.3%
4.0	3.6%	22.2%	32.8%	59.7%	42.1%	89.4%
0.0	0.0%	6.4%	8.7%	16.3%	27.1%	90.5%

	Amk (µg/mL)					
2637.014 (µM)	0	8	16	24	32	64
24.0	0.0%	59.5%	72.0%	74.2%	76.7%	77.1%
16.0	0.0%	55.5%	64.0%	62.9%	56.3%	87.1%
8.0	0.0%	39.7%	42.0%	51.4%	52.9%	83.8%
4.0	0.0%	19.3%	30.3%	58.2%	39.9%	89.0%
0.0	0.0%	6.4%	8.7%	16.3%	27.1%	90.5%

Figure S6. Checkerboard assays. The left column shows the measured values (medians %). The right column shows the adjusted values after removing any antimicrobial activity exerted by the testing compounds. Adjustment was carried out as described in Materials and Methods.

Figure S7

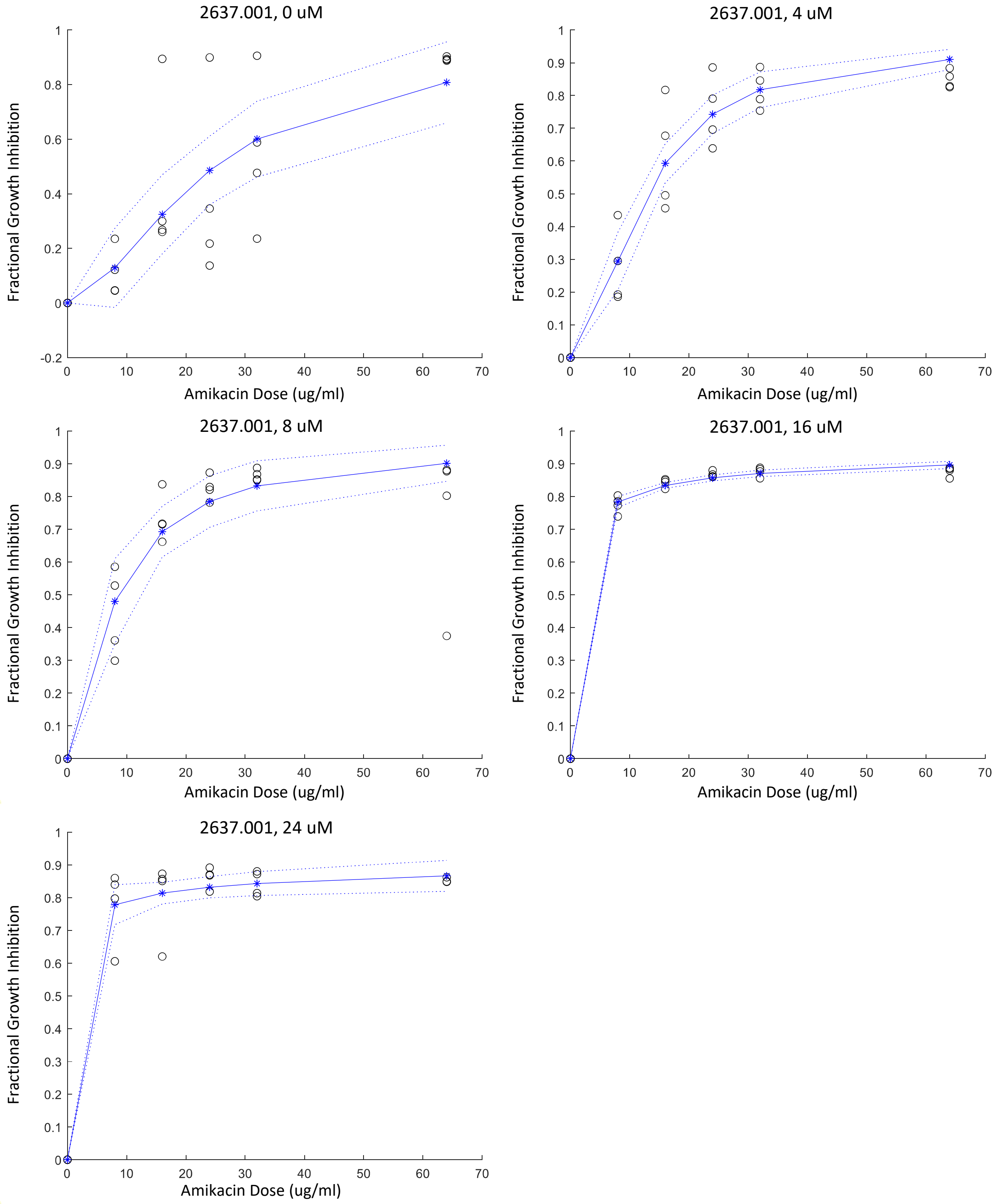
2637.001, 0 uM
2637.001, 4 uM
2637.001, 8 uM
2637.001, 16 uM
2637.001, 24 uM
Fractional Growth Inhibition
Amikacin Dose (ug/ml)

Figure S7 continued

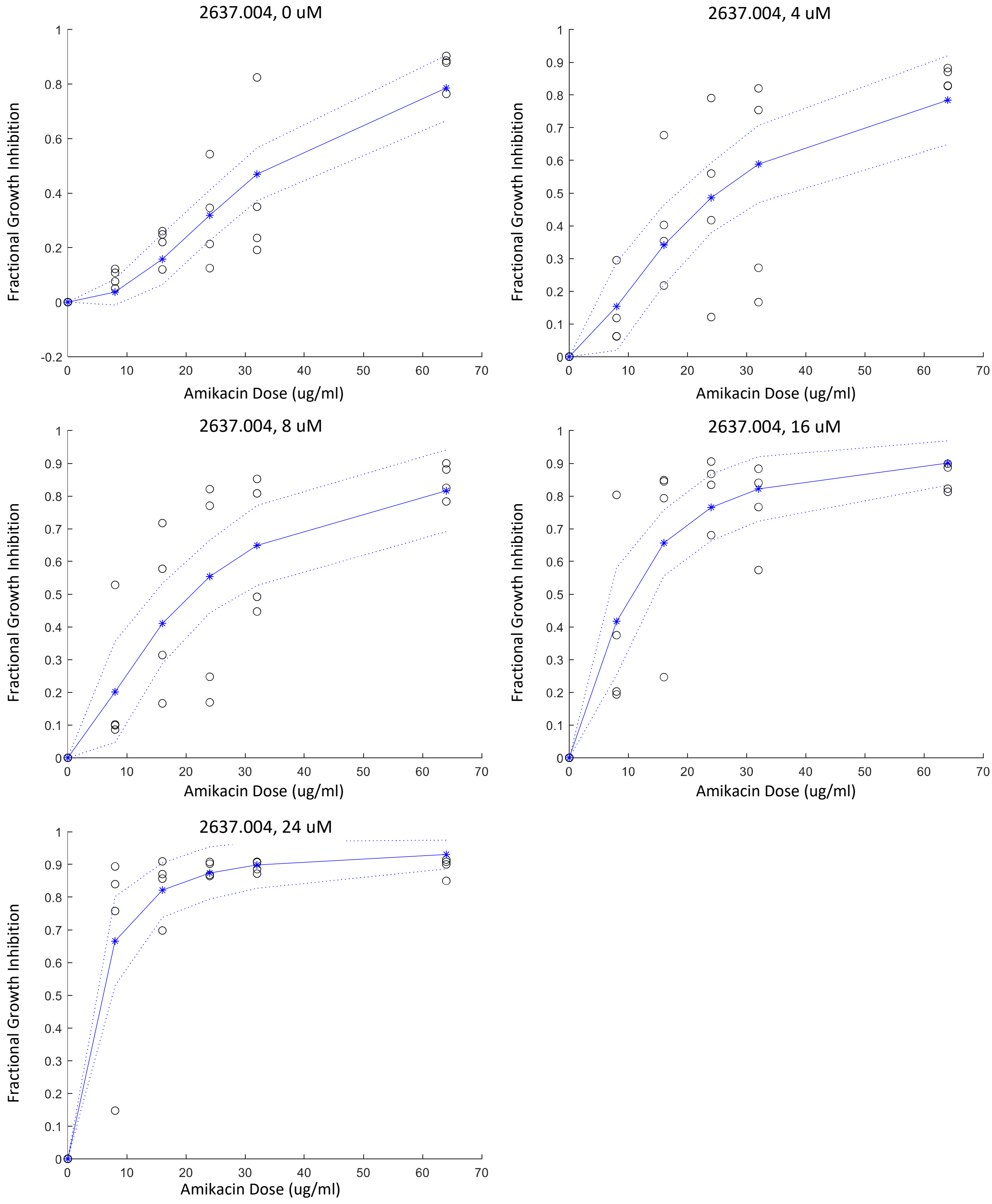

Figure S7 continued

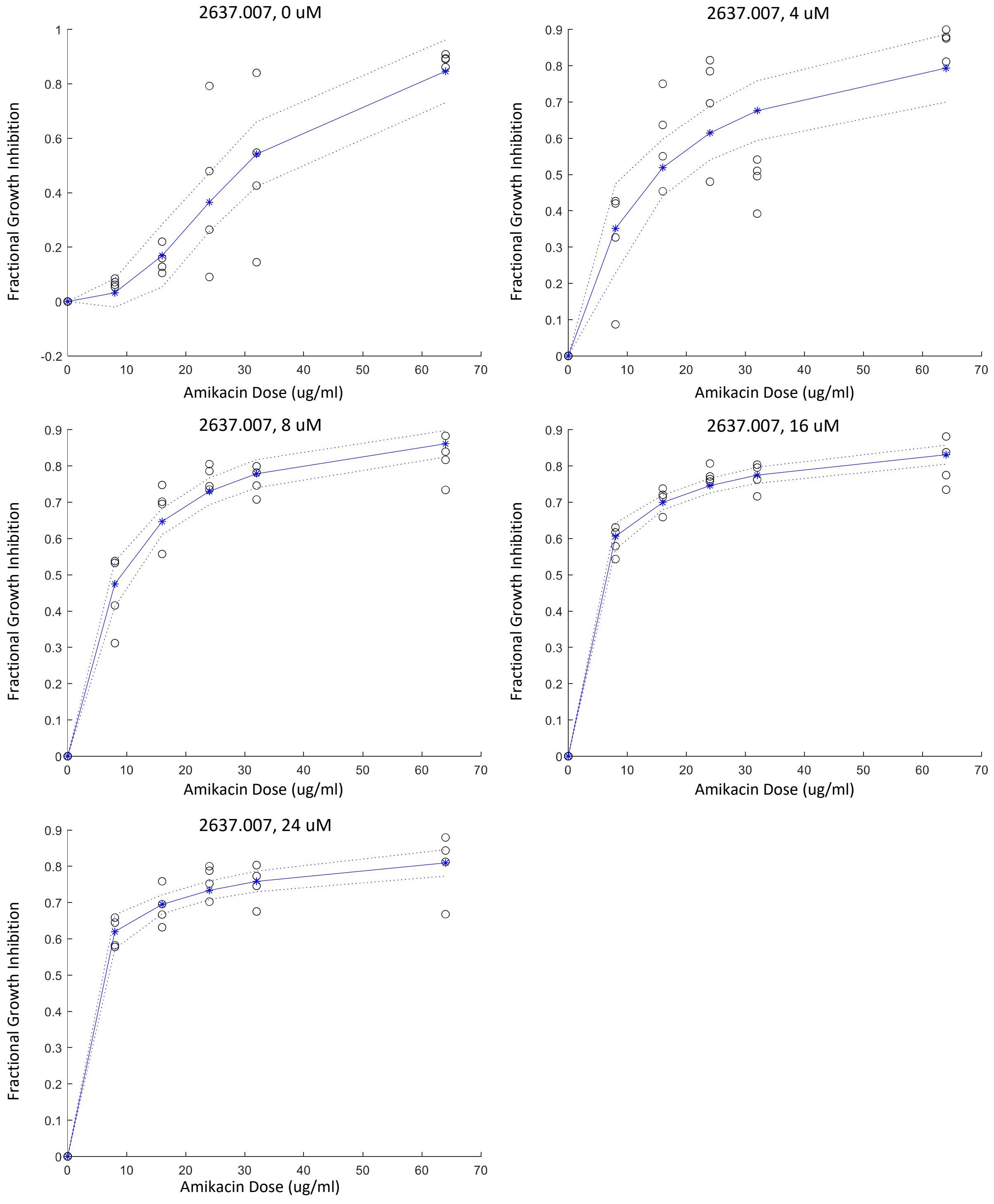

Figure S7 continued

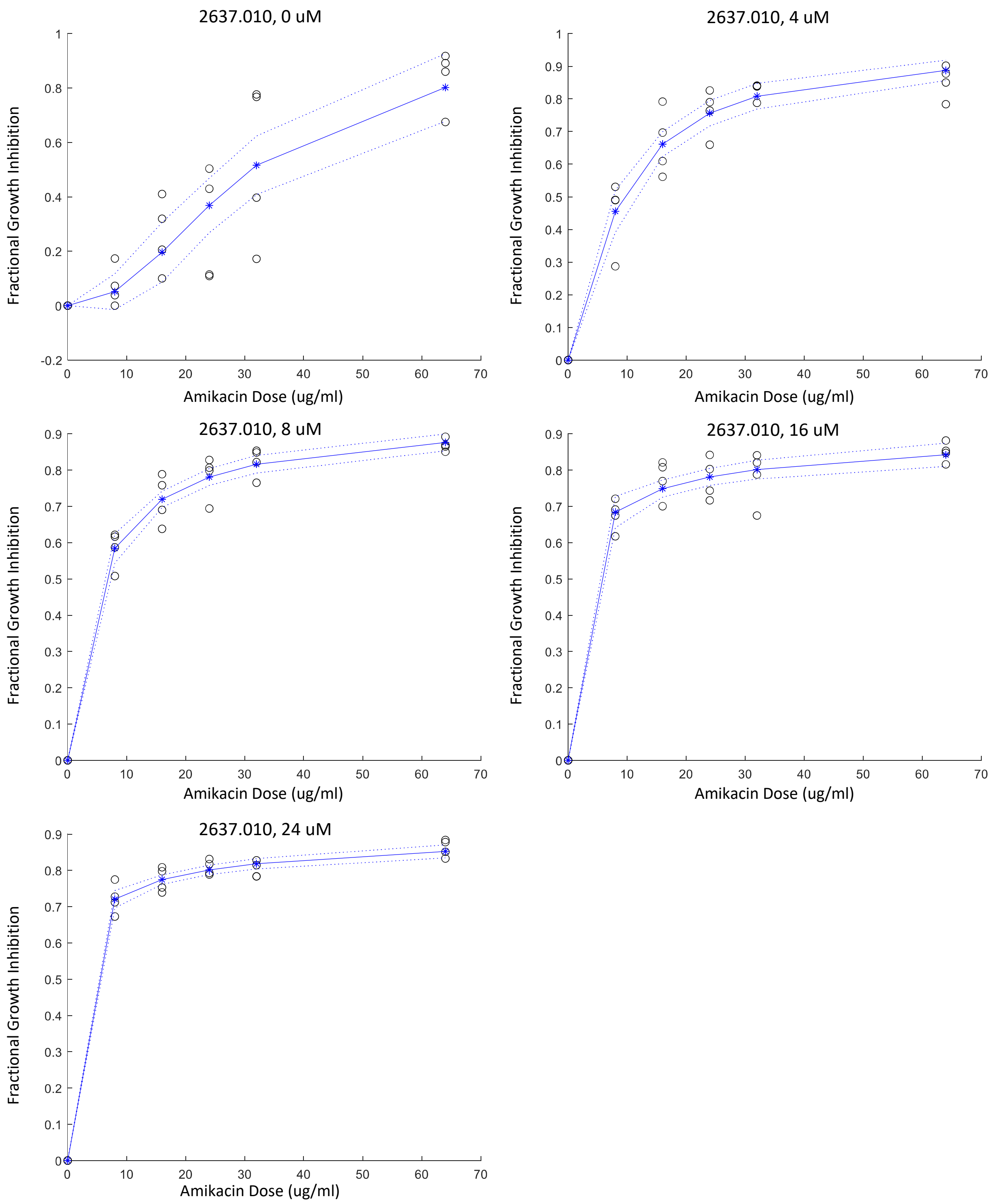

Figure S7 continued

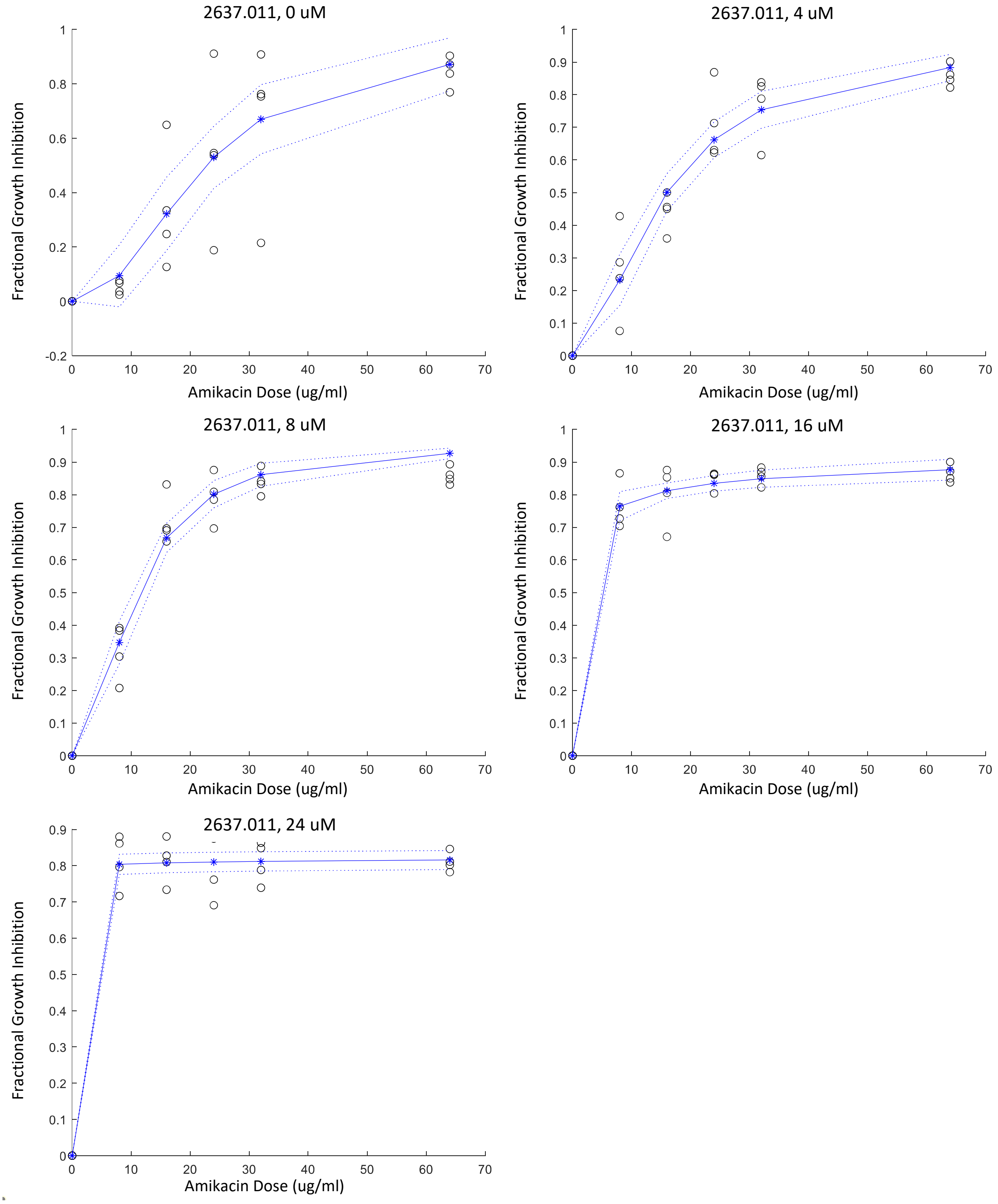

Figure S7 continued

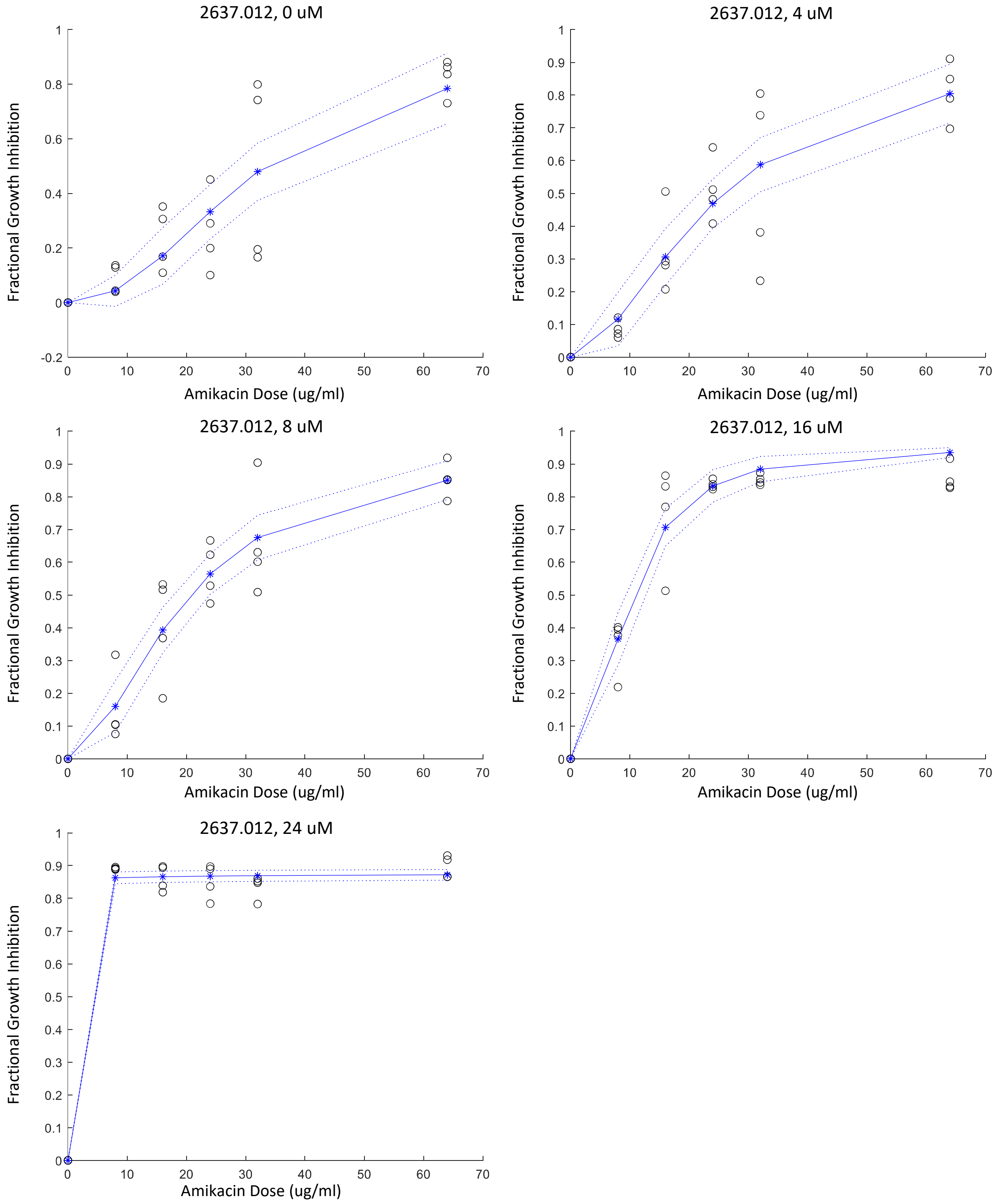

Figure S7 continued

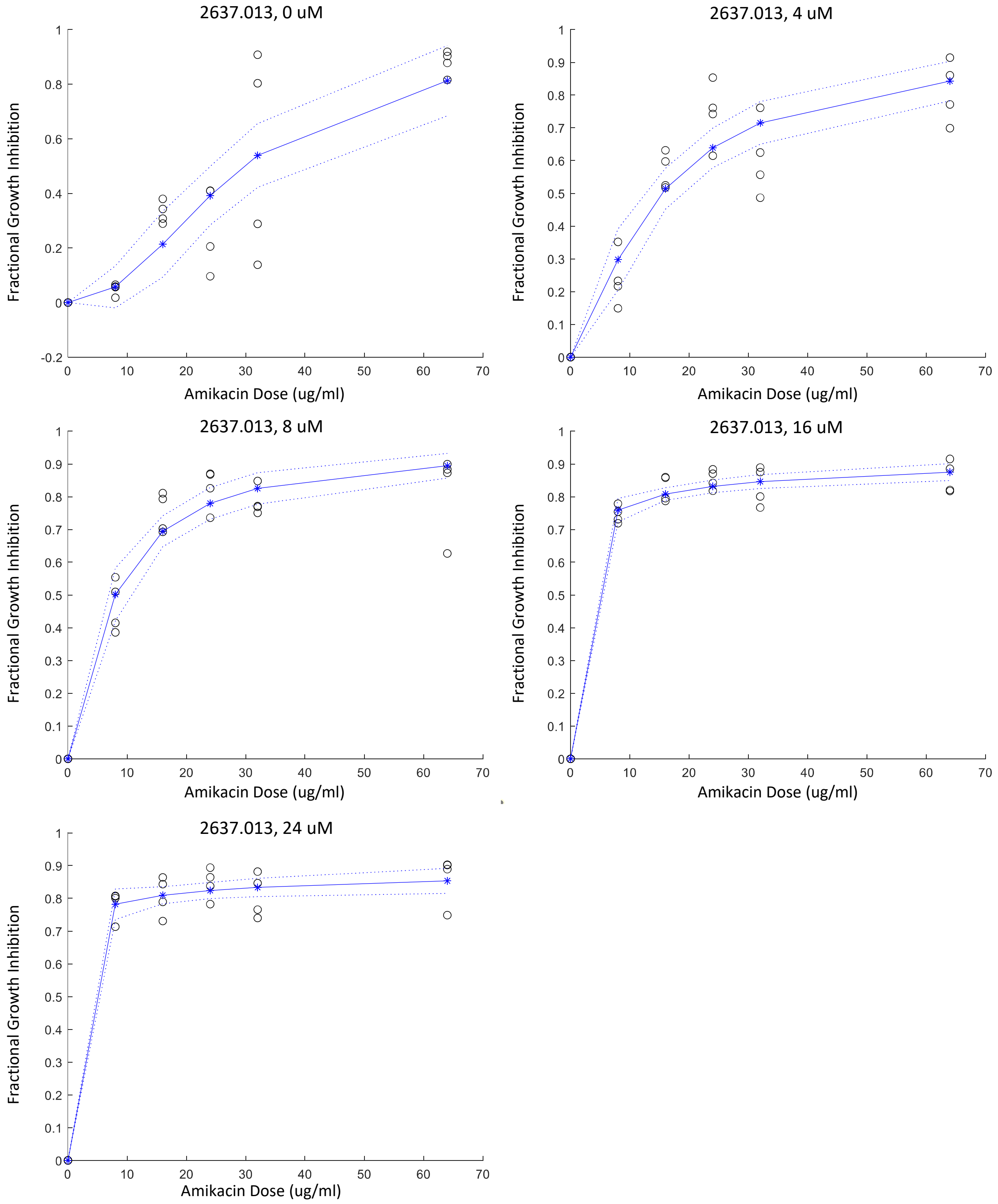

Figure S7 continued

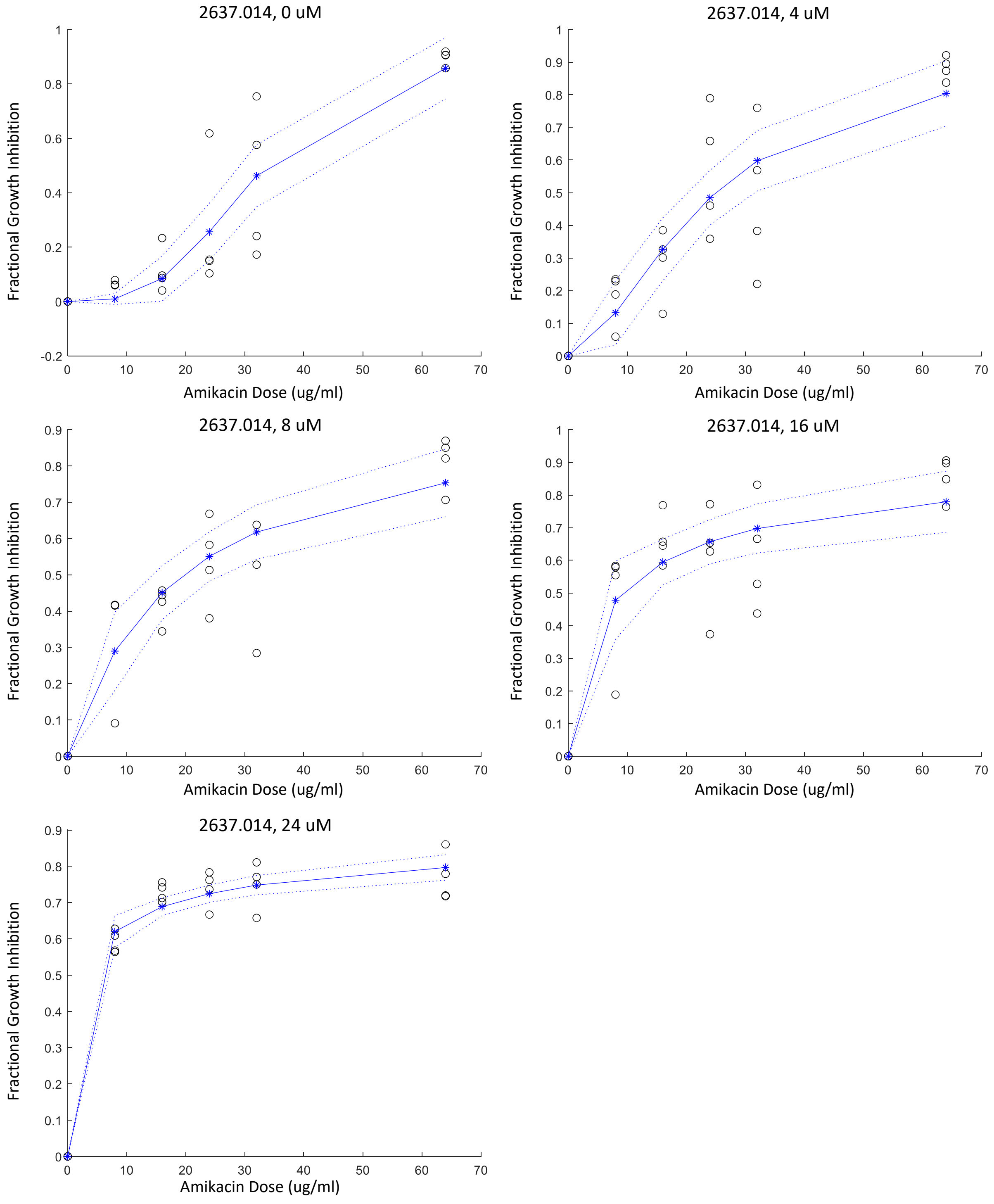

Figure S7 continued

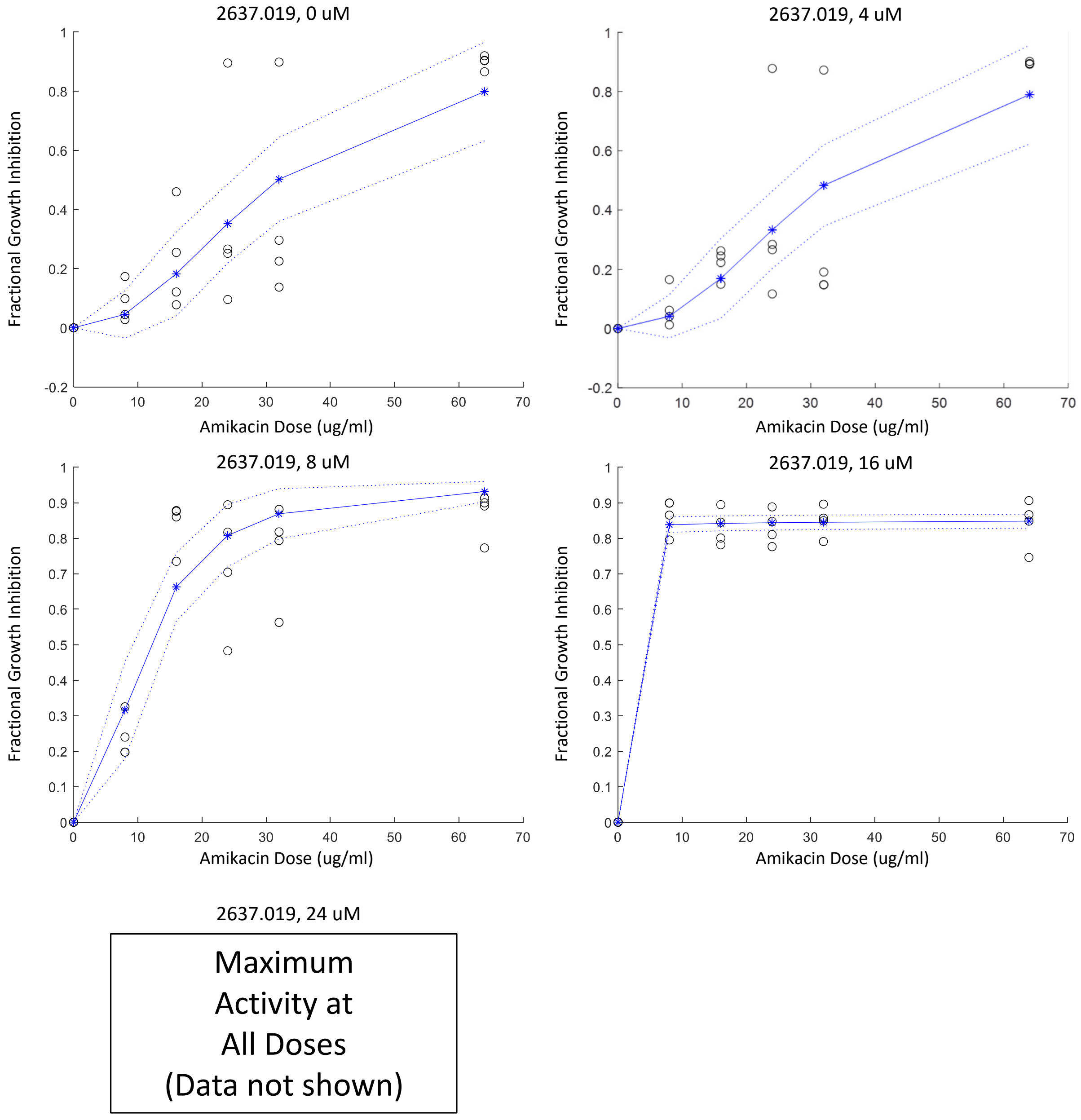

Figure S7 continued

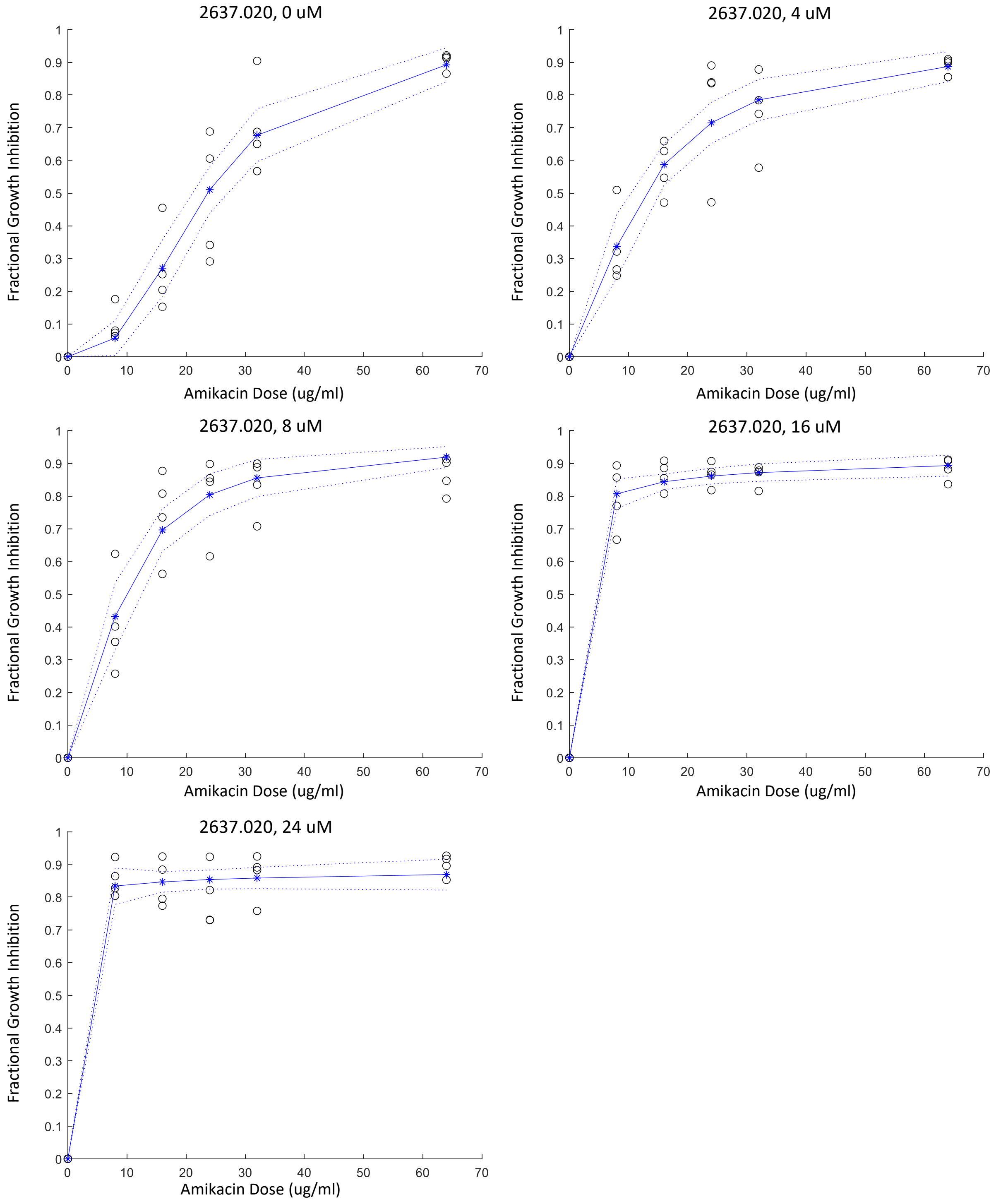

Figure S7. Fractional inhibition curves for all compounds tested in the checkerboard assays. Blue curves represent the nonlinear least squares regression line, with 95% confidence envelope dotted.
